# Global land subsidence mapping reveals widespread loss of aquifer storage capacity

**DOI:** 10.1038/s41467-023-41933-z

**Published:** 2023-10-04

**Authors:** Md Fahim Hasan, Ryan Smith, Sanaz Vajedian, Rahel Pommerenke, Sayantan Majumdar

**Affiliations:** 1 Department of Civil and Environmental Engineering, Colorado State University, Fort Collins, CO 80523 USA; 2 Department of Geosciences and Geological and Petroleum Engineering, Missouri University of Science and Technology, Rolla, MO 65409 USA; 3 Division of Hydrologic Sciences, Desert Research Institute, Reno, NV 89512 USA

**Keywords:** Hydrology, Natural hazards

## Abstract

Groundwater overdraft gives rise to multiple adverse impacts including land subsidence and permanent groundwater storage loss. Existing methods are unable to characterize groundwater storage loss at the global scale with sufficient resolution to be relevant for local studies. Here we explore the interrelation between groundwater stress, aquifer depletion, and land subsidence using remote sensing and model-based datasets with a machine learning approach. The developed model predicts global land subsidence magnitude at high spatial resolution (~2 km), provides a first-order estimate of aquifer storage loss due to consolidation of ~17 km^3^/year globally, and quantifies key drivers of subsidence. Roughly 73% of the mapped subsidence occurs over cropland and urban areas, highlighting the need for sustainable groundwater management practices over these areas. The results of this study aid in assessing the spatial extents of subsidence in known subsiding areas, and in locating unknown groundwater stressed regions.

## Abstract (web summary)

Groundwater overdraft can lead to land subsidence and groundwater storage loss. Here, the authors develop a machine learning-based method to map subsidence globally, explore subsidence drivers, and identify regions under high groundwater stress.

## Introduction

Excessive groundwater pumping can cause depletion, loss of aquifer storage capacity, arsenic contamination, saltwater intrusion, and infrastructure damage^1–3^. Despite its importance, many regions of the world with intensive groundwater withdrawals and storage loss are poorly monitored. In absence of spatially dense monitoring networks, publicly available in situ data, and uniform monitoring strategies, it is challenging to quantify groundwater storage loss. To address such data gaps, remote sensing techniques have been used to develop global scale datasets that measure proxies of or drivers for groundwater storage change. However, no current remotely sensed dataset provides a direct estimate of available groundwater storage and storage loss.

One of the most visible and harmful effects of groundwater depletion is land subsidence, which is caused by compaction of aquifer materials following the loss of pore pressure^4^ and can cause irreversible loss of aquifer storage capacity^5^. Estimating the amount of subsidence can be used to quantify storage loss in unconsolidated confined aquifer systems^1^. In-situ measurement methods for quantifying subsidence exist; however, they are spatially far too sparse to be used in accurate subsidence estimation at regional to global scales. Interferometric Synthetic Aperture Radar (InSAR) observations have been shown to be a reliable source of subsidence data, providing ~1 cm accuracy at a fine spatial resolution of ~100 m^6^, and have been used to monitor groundwater storage depletion in many aquifer systems^7–9^. Despite that, processing InSAR data is computationally expensive and can be challenging to interpret in the presence of tropospheric or ionospheric noise^10,11^; therefore, InSAR-based groundwater studies have been limited to the local or regional level. This hampers our ability to understand the state of subsidence and loss of aquifer storage capacity in regions outside the scope of these studies.

Process-based models provide another method for developing global estimates of groundwater availability and storage change^12,13^ Nevertheless, a global model of land subsidence has not been produced to date. Such an effort would require extensive geomechanical and hydrogeologic datasets, and knowledge of the temporal evolution of head changes driving non-linear subsidence processes^1,5^, which are not available at the global scale. However, remote sensing and global model-based datasets offer estimates of some of the drivers of subsidence and can be useful for predicting subsidence with statistical or data science approaches. While some studies have attempted to predict loss of aquifer storage capacity from subsidence at regional scales^1,14^, and subsidence susceptibility globally^15^, no existing study has quantified the magnitude of subsidence and associated groundwater storage loss globally.

In this study, we present a machine learning method to map pumping-induced land subsidence at a high spatial resolution (~2 km) on a global scale, using remote sensing and model-based hydrologic, land use, climatic, and geologic datasets. We trained the model^16^ with an extensive InSAR and Global Navigation Satellite System (GNSS)–based land subsidence dataset. Our method produces subsidence estimates in <1 cm/year, 1–5 cm/year, and >5 cm/year classes. This study is a global-scale endeavor to map subsidence across ranges of magnitude and related groundwater storage loss at high (~2 km) resolution, in addition to exploring drivers of land subsidence. Such a study is crucial from the perspective of climate change, population growth, and relative sea level rise, which threaten to increase water scarcity, coastal flooding, and saltwater intrusion. The resulting global subsidence map can be interpreted as a first-order map of global aquifer storage loss due to loss of porosity, which is the primary mechanism for groundwater storage loss in confined alluvial basin aquifers^1^. In addition, the machine learning model^16^ provides a global subsidence probability map (Supplementary Figs. 2 and 3, Supplementary Discussion 4) which is critical in identifying regions that are likely to experience subsidence. Using the model results, we are able to generate country level statistics of loss of aquifer storage capacity caused by subsidence, identifying countries with aquifers under the highest threat and putting groundwater stress in a global context.

## Results and discussion

### Global subsidence map

**Fig. 1:**
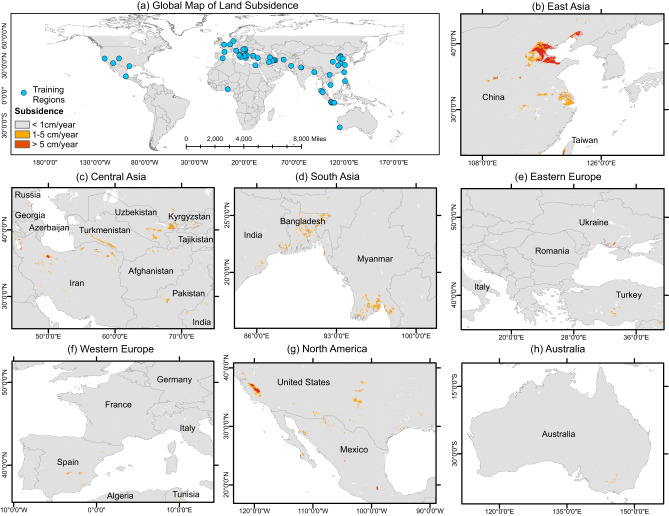
Groundwater withdrawal induced global land subsidence predicted by the random forest model. The model has been trained with Interferometric Synthetic Aperture Radar (InSAR)-derived subsidence data for 47 regions (blue dots) and a Global Navigation Satellite System (GNSS)-based coastal subsidence dataset to generate the **a** Global map of subsidence. 1–5 cm/year and >5 cm/year are considerable subsidence classes, and <1 cm/year is the nominal or no subsidence class. The model predicts significant subsidence across the globe that covers regions in **b** East Asia, **c** Central Asia, **d** South Asia, **e** Eastern Europe, **f** Western Europe, **g** North America, and **h** Australia. Source data are provided as a Source data file.

A random forest algorithm-based machine learning approach has been used in this study to generate a high-resolution (~2 km) global map of land subsidence. The global subsidence model^16^ was designed to predict subsidence in three classes: <1 cm/year, 1–5 cm/year, and >5 cm/year, with the <1 cm/year class considered as the nominal or zero subsidence class. It was trained with InSAR-based subsidence datasets from 47 regions and a GNSS-based coastal subsidence data^17^ of the world; using hydrologic, land use, and geologic datasets as input variables/predictors that are estimates and proxies of principal drivers of land subsidence. Figure 1 (see Supplementary Fig. 1. for the whole map) shows the global map of subsidence, focused on regions with high subsidence signatures, mapped by our model. It should be noted that the model developed in this study is designed to only estimate subsidence related to aquifer system compaction from groundwater pumping; therefore, the total subsidence estimates over some regions, which are undergoing subsidence from other sources, may not match.

The model maps a considerable amount of subsidence in countries of East Asia: China, Taiwan, Vietnam, and the Philippines (Fig. 1 and Supplementary Fig. 1). In China, intensive irrigation activities have been mapped over the North China Plain aquifer by global irrigation mapping studies^18^. Major cities like Beijing, Shanghai, Wuhan, Xian, and Tianjin are in or nearby this region and are heavily dependent on groundwater to support agriculture and urban needs^8,19–22^. Our map shows a high subsidence signature in this whole region indicating significant groundwater storage decline. Countries in South, Central, and middle-East Asia, such as Bangladesh, Myanmar, India, Pakistan, Indonesia, Iran, and Turkey have areas of high subsidence signals as well. These predictions are in line with recent InSAR based groundwater studies for these regions^9,23–31^. Our model also predicts subsidence in irrigated and urban regions over Afghanistan, Turkmenistan, Uzbekistan, Azerbaijan, and Syria (Supplementary Fig. 1), where no previously published land subsidence studies due to groundwater withdrawal were available.

In Europe, recent studies have shown subsidence occurring in Spain, Italy, and England^32–34^ with low magnitude of less or marginally higher than 1 cm/year. Our model categorizes the majority of subsidence in Europe as <1 cm/year. Vertical land movement data from GNSS^17^ and European Ground Motion Service (EGMS) over Italy, Spain, France, Hungary, and Greece also show deformation lower than 1 cm/year, which is too low for our model to predict; however, subsidence of such magnitude can be significantly damaging in coastal areas, compounded with the impacts of sea level change. The map predicts subsidence between 1–5 cm/year, primarily due to groundwater irrigation, in Albacete and Ciudad Real province, and Alto Guadalentín basin in Spain. Comparison of model prediction in Albacete and Ciudad Real province with the EGMS data show that the model overestimates subsidence in these regions, possibly due to uncertainty in representing groundwater irrigation estimate in the model. Some 1–5 cm/year deformation signals have also been mapped in the dominantly agricultural region near Po Delta, Italy which were validated by the EGMS data. The model also predicts >1 cm/year subsidence in irrigated regions near the coast of Ukraine.

In North America, Fig. 1 shows considerable subsidence in California, Texas, Arizona, and central Mexico, which follows historic subsidence observed in these regions^5,7,35^. Subsidence in California and Arizona is due to excessive groundwater irrigation^35,36^, while urban dependency on groundwater is responsible for deformation in Houston and Mexico City^7,37^. The model overestimates subsidence in some regions of the heavily pumped High Plains Aquifer^38^ where a recent study has estimated <1 cm/year subsidence^39^.

InSAR studies in Africa^40,41^ have estimated less than 1 cm/year subsidence in the Nile river delta and in several coastal cities, such as Lagos, Banjul, Mombasa, and Mogadishu. The model’s predictions show similar subsidence rates in these areas. The model predicts 1–5 cm/year subsidence in Morocco, Algeria, and Tunisia over irrigated lands (Supplementary Fig. 1). In Australia, subsidence <1 cm/year has been detected in Perth and irrigation dependent Murray-Darling basin, with few locations undergoing higher than 1 cm/year subsidence^42^. Our map detects 1–5 cm/year subsidence in the Murray-Darling basin region which might be happening in the alluvial aquifers consisting of clay and silt^43^. In the South American continent, >1 cm/year subsidence has been mapped over small, irrigated regions in Argentina and Peru. In Bolivia, >5 cm/year subsidence has been predicted over groundwater dependent cities^44,45^ of Oruro and Cochabamba.

While the model generally shows good agreement with observed subsidence data, it overestimates subsidence in some regions, which happens due to uncertainty associated with the input variables. A full discussion of input variable uncertainty is provided in Supplementary Discussion 3 and summarized here. We developed a “confining layer” input variable to represent the presence of depositional lacustrine and marine confined aquifer conditions using a digital elevation model (DEM). Although this dataset has been validated successfully for the major aquifers in the United States (Supplementary Method 2), imprecise delineation of depositional settings in other regions can add some uncertainty to the model prediction. In addition, we developed the “normalized clay indicator” variable using datasets of percent clay content data at 200 cm^46^ depth and average unconsolidated material thickness^47^ (detail in the “Methods” section). While the normalized clay indicator is an effective proxy for the presence of thick clays at greater depths, it is limited by the shallow depth of clay content used and thus adds uncertainty to our model.

### Drivers of land subsidence and groundwater storage loss

In confined (pressurized) aquifers, the aquifer matrix remains saturated even as the pressure head in the aquifer drops, and storage loss occurs. The two mechanisms for storage loss in confined aquifers are loss of pore space in the aquifer due to consolidation, and expansion of water^48^. In confined aquifers that are unconsolidated (which are commonly the most productive^49^), consolidation accounts for the vast majority of storage change^5^. Thus, subsidence in a confined or semi-confined aquifer system is a first-order estimate of total aquifer storage loss^1^.

Land subsidence is driven by several factors, including aquifer skeletal specific storage (${S}_{{sk}}$), thickness of compressible sediments ($b$), change in hydraulic head ($\varDelta h$) due to pumping and recharge, and the consolidation history of the layer experiencing subsidence. More background on the mechanism of land subsidence has been described in Supplementary Note 1. For local and even for some regional areas, these datasets are available and can be coupled to estimate land subsidence. However, data scarcity, heterogeneity of collected data, and coarse resolution of available data make it difficult to compile all required datasets at a global scale. Global coverage of remotely sensed datasets can bridge this void by providing estimates of variables, such as precipitation, evapotranspiration (ET), soil moisture, and total water storage (TWS) data, in areas with heavy groundwater exploitation and can give an indication of hydrologic change. TWS anomaly data from the Gravity Recovery and Climate Experiment (GRACE) satellite along with other hydrologic variables of coarse scale have been used in multiple studies to model groundwater storage change at regional and global scales^50,51^. However, GRACE has an effective spatial resolution no better than about 100,000 km^2^ at mid-latitudes^52^. An analysis of subsidence training data with GRACE TWS trend (over 2013–2017) revealed that ~36% subsidence training pixels fall in regions where TWS has a positive trend. TWS comprises both surface and groundwater fluxes, and a GRACE storage trend over a 100,000 km^2^ region can have a positive trend even if groundwater storage is in decline within a 4 km^2^ pixel within the larger GRACE region. Due to the coarse resolution and to avoid biases in model training, GRACE data was not incorporated in the model. Rather, InSAR processed land subsidence data can function as a proxy of groundwater storage change in aquifers, as discussed in the previous section. Our model assimilates multiple hydrologic and land use variables which can be related to groundwater storage change. Geologic variables, such as normalized clay indicator and existence of confining units, have also been used in the model as proxies of aquifer properties, and they represent the presence of fine sediments units in the subsurface which is a major driver of subsidence.

**Fig. 2:**
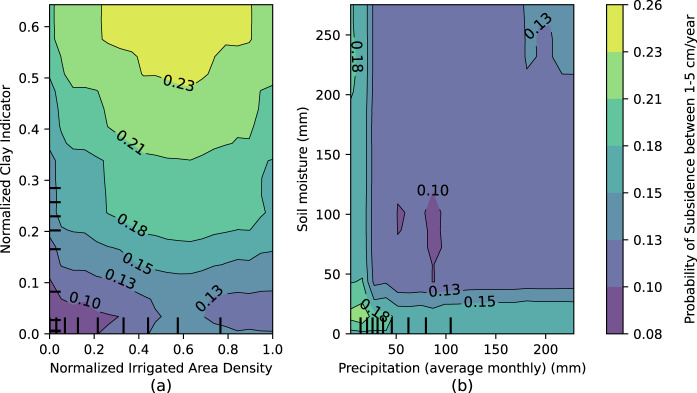
Two-way partial dependence plots of input variables. The plots represent contributions of input variable combinations **a** Normalized Irrigated Area Density and Normalized Clay Indicator and **b** Precipitation and Soil Moisture in predicting 1–5 cm/year subsidence. Warmer color indicates higher subsidence probability. The values in both axes have been plotted between 1st to 99th percentile as the model’s response (in predicting subsidence) to the variables is more evident within this range. Normalized clay indicator and normalized irrigated area density are proxy variables that indicate the presence of fine sediments and groundwater irrigation density in the model, respectively, whereas soil moisture and precipitation, along with other hydrologic fluxes, represent water balance indicating areas with groundwater depletion.

To assess if the input variables realistically illustrate the physical processes in the machine learning model, we analyzed the Partial Dependence Plots (PDP). Two-way PDP plots in Fig. 2 represent the contributions of input variables in model predictions of subsidence of the 1–5 cm/year class. Supplementary Fig. 4 contains individual PDP for some of the key variables of the model.

The input variables added to the model were direct measurements or proxies of principal drivers of land subsidence and groundwater withdrawal. The presence of clay and fine-grained confining units in confined or semi-confined aquifers are the major impetus of inelastic, high-magnitude subsidence. In addition, high density of irrigated agriculture in an area indicates higher probability of groundwater being used for irrigation in the presence of limited or no surface water resources. Land use with high groundwater irrigation is prone to subsidence if the water is being drawn from the fine-grained sediments in the subsurface. Figure 2a illustrates the model’s ability to understand this relationship between normalized irrigated area density and normalized clay indicator (values ranging from 0 to 1, higher values indicate higher presence of irrigation density and clay), as the response shows higher probability of subsidence with high irrigated area density and normalized clay indicator.

The hydrologic variables represent water balance in the model indicating regions with groundwater depletion. Irrigation demands more groundwater in arid and semi-arid regions where precipitation is lower than evapotranspiration and surface water sources are scarce. In such regions, excessive groundwater pumping depletes groundwater storage which can lead to subsidence under favorable geologic conditions. Soil moisture is regarded as the most important variable by our model (Supplementary Fig. 8) because it is an effective indicator of the hydrologic conditions (sustained high temperature and low precipitation) that lead to subsidence if other principal drivers of subsidence are present (detailed discussion in Supplementary Discussion 2). Low soil moisture from a land surface model that does not account for irrigation, such as the one used in this study, indicates high irrigation water demand in croplands. Our model predicts higher subsidence probability in regions with low precipitation and soil moisture (Fig. 2b). The interaction between variables in predicting subsidence, as shown in the PDP plots, confirms that the response of land use, geologic variables, and hydrologic fluxes in the model are realistic. Supplementary Discussion 1 and 2 discuss the interaction between the input variables and their interpretation.

### Country statistics of subsidence and groundwater storage loss

**Fig. 3:**
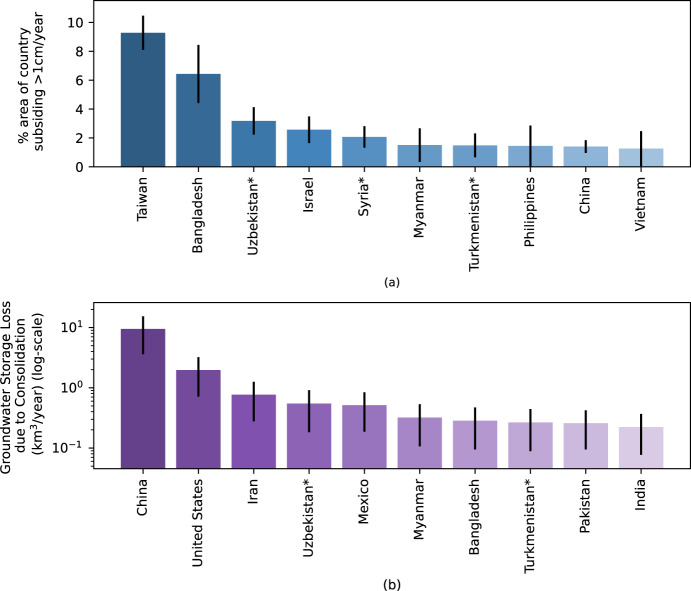
Rankings of country-level statistics of subsidence of magnitude >1 cm/year for the top 10 countries. **a** shows countries with the highest percentage of subsidence with respect to their country area. The error bars represent the standard deviation of the mean (standard error) of % area subsiding estimates. **b** shows countries with the highest groundwater storage loss, predicted by our model. China, the United States, and Iran account for the majority of permanent aquifer storage loss due to consolidation. The error bars represent the upper and lower bounds of groundwater storage loss estimates. In total, we have estimated an average ~17 km^3^/year (a first-order estimate) of confined aquifer storage loss globally (lower and upper bounds ~11.5 km^3^/year and ~22 km^3^/year, respectively). Countries with asterisks are where no previously published land subsidence studies due to groundwater withdrawal were available. In both plots, Source data are provided as a Source data file.

Results from the global subsidence model were used to generate country-level statistics of subsidence and groundwater storage loss to get a global perspective of groundwater stress. Figure 3a shows 10 countries with the highest percentage, by area, of land that is experiencing 1 cm/year of subsidence or greater. Our model has mapped significant subsidence over small island nations like Taiwan and the Philippines along with many other coastal regions, and over semi-arid and arid climates in Uzbekistan, Azerbaijan, Armenia, and Turkmenistan (Supplementary Fig. 1), where studies have reported high groundwater uses^27,53–56^.

Subsidence is not limited to arid regions; it has been mapped in humid climates, including Bangladesh, India, Vietnam, and Indonesia (see Supplementary Fig. 10 for more rankings), indicating their dependency on groundwater despite having high precipitation supply. Additionally, the model results were used to estimate a permanent global groundwater storage loss value of ~17 km^3^/year due to aquifer consolidation. China, the United States, and Iran account for the majority of this loss (Fig. 3b). Overall, a comparative assessment between the percentage area affected by subsidence and total aquifer storage loss is required to understand the comprehensive groundwater stress scenario of a particular country.

### Land uses driving subsidence and implications in planning

Comparison of mapped subsidence with the Moderate Resolution Imaging Spectroradiometer (MODIS) land use product reveals that most of the predicted subsiding regions are on either croplands or urban areas (~60.5% are on croplands, ~12.5% are on urban and built-up lands), ~19% are on vegetated, uncultivated regions, and 8% on other land cover types. The MODIS land use product reports an overall accuracy of 75%^57^; therefore, there is a possibility of intermixing between the vegetation and cropland classes. Vegetated lands sometimes exists in the vicinity of croplands and might even be periodically used for agriculture, which may explain the high predicted subsidence rates in vegetated areas. The high probability of subsidence associated with increasing irrigation and population density (Supplementary Fig. 4) represents the model’s ability to understand the relation between long-term subsidence and groundwater use in those regions. Moreover, subsidence predictions are high (~75%) in arid and semi-arid climates where climatic water deficit leads to higher groundwater dependency.

In regions where groundwater usage from aquifers, especially from confined and semi-confined layers, is significantly more than the volume of recharge over a long period, inelastic subsidence causes permanent aquifer storage loss due to consolidation^5^. This indicates that in the predicted subsiding locations, groundwater storage is permanently declining. Our model overestimates subsidence in some regions due to uncertainty in the input variables. Despite that, the generated map provides a first-order global estimate of subsidence due to groundwater overdraft. Comparison with documented subsidence locations and global groundwater studies (Supplementary Discussion 5 and Supplementary Fig. 13) shows that the machine learning model was able to reveal the true spatial extent of subsidence in many regions. These subsiding areas may continue to experience subsidence, and potentially experience an increase in subsidence magnitude and area, if water use practices are not modified. We also identified subsidence in irrigated and populated locations where groundwater related subsidence has not been studied and reported before. These regions are undergoing groundwater stress, and it is essential to develop effective, long term aquifer monitoring strategies to understand the true dynamics of groundwater resources in the affected regions. Additionally, regional studies incorporating InSAR data analysis should be undertaken for the mapped subsidence risk areas. Such efforts will help to formulate appropriate groundwater use, recharge, and long-term action plans for aquifer sustainability.

## Methods

### Processing input hydrologic and land use datasets

Input variables (predictors) of this model include remotely sensed and model-based global gridded datasets^58^ that are proxies of principal hydrologic, geologic, and anthropogenic processes that drive land subsidence. Supplementary Table 1 shows a comprehensive list of all input datasets used in the model along with their original spatial resolution and sources. Depending on the original resolution, the datasets^58^ were downscaled/upscaled to a resolution of 0.02 deg (~2 km) using the “nearest neighbor” algorithm to achieve a uniform grid size.

Direct, global estimates of groundwater resources are not available at the 2-km scale of our model from remote sensing sources. However, other water balance components such as precipitation, soil moisture and evapotranspiration are available, and correlate with withdrawals and recharge, important fluxes^59^ whose relative magnitude is one control on subsidence^60^. We added these variables to account for these crucial water balance components.

A global irrigation area dataset Meier et al.^18^ at ~1 km resolution was used in this study as one of the land use datasets. This dataset was developed by combining remote sensing datasets and downscaled statistics-based irrigated area data from an irrigation dataset^61^ produced by the Food and Agriculture Organization of the United Nations (FAO). A gridded population dataset of ~1 km resolution^62^ was included in the model to capture subsidence in populated areas occurring from aquifer pumping. A Gaussian filter was applied to both of the irrigated area and population datasets to add a smoothing effect that accounts for groundwater depletion in regions adjacent to aquifer pumping and to remove noise. The Gaussian filter normalized the datasets within an interval range of 0–1, where larger values represent higher density of respective land use class and vice versa. Supplementary Method 1 describes more about input dataset processing.

### Processing input geologic datasets

Existence of fine sediments is a major geologic factor for inelastic subsidence^63^. Global geologic datasets indicating the presence of fine sediments is not readily available; therefore, existing global geologic datasets have been modified to form proxy variables that indicate the presence and relative magnitude of fine sediments in the subsurface.

High-resolution (250 m) percent clay content data at 200 cm below ground surface, generated from a soil information based machine learning model^46^, was multiplied with an average unconsolidated material thickness dataset (~1 km resolution)^47^. This product was normalized to form the “normalized clay indicator” dataset, a proxy dataset representing presence of clay in the subsurface.

Because the presence of a confining layer has a pronounced impact on the relationship between pumping and subsidence, a dataset indicating the likely presence or absence of a confining layer was produced as part of this study (Supplementary Fig. 11). This confining layer dataset was derived based on the depositional environment of basins and was produced using a globally available DEM from the Shuttle Radar Topography Mission (SRTM)^64^. The premise used in creating this dataset is that regions that are likely to have had lacustrine or oceanic depositional environments over the past several hundred thousand years are also likely to have extensive clay layers, which confine the aquifer and result in more subsidence from groundwater withdrawals. We validated our confining layer model using major aquifers in the United States as defined by the Groundwater Atlas of the United States^65^, because this provides an extensive, geo-referenced map of aquifers with major confining layers. Supplementary Table 2 shows a summary of each major aquifer, along with the percent area of each aquifer that is estimated to have a confining layer present based on the methods outlined above. Detail discussion on developing this dataset along with its validation method have been presented in Supplementary Method 2.

### Assembling land subsidence dataset for model training

Supervised machine learning algorithms require a training dataset to establish relationships between training data and input variables. Average vertical land subsidence rates (units in cm/year), in areas where significant groundwater pumping has been recorded historically, were used as training data in the machine learning model. Subsidence data were collected for 47 regions of the world from InSAR sources. In addition, a global coastal subsidence dataset was obtained from a GNSS-based study^17^. In this study, deformation data was gathered in three ways: processed by the authors; obtained as georeferenced, pre-processed data from public agencies of the United States and EGMS; and georeferenced from published studies.

We processed subsidence data in regions where no recent (2013 and later) subsidence data were available to the authors’ knowledge in the published literature: Quetta Valley, Pakistan; Qazvin, Iran; and San Luis Valley, Colorado, United States. Our initial model revealed significant subsidence in the Hebei and Hefei regions of China; therefore, we processed InSAR data for these regions and included that in the training dataset. For processing InSAR, Small Baseline Subset (SBAS) InSAR time series analysis was conducted to estimate the average vertical subsidence rate in the Line of Sight (LOS) direction. LOS velocities were further decomposed into vertical and horizontal components using measurements from ascending and descending imaging geometries.

Processed, georeferenced vertical subsidence data over California and Arizona in the USA were collected from the California Department of Water Resources and from the Arizona Department of Water Resources, respectively. Similarly, processed, georeferenced vertical subsidence data over 15 regions of Europe were collected from the EGMS. Considering the substantial computational effort required in processing InSAR data, and the challenges associated with interpreting the principal subsidence cause to be related to groundwater, data was also collected from secondary sources. These sources consist of groundwater studies that used InSAR or GNSS information to determine aquifer vertical deformation. Regions where secondary sources were used include China, Indonesia, Iran, Turkey, USA, and Vietnam. A comprehensive list of these training data sources is provided in the Supplementary Table 3.

Finally, the subsidence data collected from these three sources were classified into three classes: <1 cm/year subsidence, 1–5 cm/year subsidence, and >5 cm/year subsidence. Subsidence data collected from research articles are referred to as georeferenced subsidence data in this study; however, these data are primarily based on InSAR processing. The classified subsidence data from the three processing methods were merged to form a final training dataset and resampled to a spatial resolution of 0.02 deg (~2 km). Supplementary Fig. 12 provides a framework of our modeling steps. It should be noted that <1 cm/year subsidence is considered as negligible to no subsidence class while the other classes represent medium to significant subsidence for this global study. However, subsidence of <1 cm/year values can be significantly damaging for coast-side regions due to the impact of climate change and resulting sea level rise, but predicting this level of subsidence was out of the scope of this study. Supplementary Method 3 describes how the training subsidence dataset was formed from multiple sources.

### Random forests model prediction

Variables interplaying in land subsidence have complex nonlinear relationships that can be explored using a machine learning model. In this study, random forests, a popular tree-based ensemble learning algorithm, was used to incorporate the input variables to predict land subsidence in three classes. Random forests algorithm performs with high efficiency without input variable scaling^66^, so datasets with values in varying units can be assimilated in such a model without issues.

Random forests employ techniques like bootstrap aggregating (bagging) of training samples and random splitting of input features to reduce variance, thus minimizing model overfitting^67^. Ensemble results of multiple trees are summarized by majority voting (in a classification model) to produce the final model outcome. The model’s key hyperparameters were optimized (detail in Supplementary Table 4), to improve model accuracy and avoid overfitting, using a random search 10-fold cross-validation approach. The optimized model has hyperparameter values of n_estimators = 300, max_depth = 14, max_features = 7, min_samples_split = 7, and min_samples_leaf = 1^−5^, which resulted in a macro F1-score of 0.83 on the test set.

Random forests model creation requires a primary training dataset (also referred to as response variable), in this case, land subsidence data. Machine learning models learn the relationship between input variables using the response variable. To create the training dataset for our model, pixels containing a land subsidence classification (<1 cm/year, 1–5 cm/year, or >5 cm/year) were matched to the input variables at the co-located pixel. The resulting dataset is referred to as the original training dataset of the model. This original training dataset was randomly split into train and test sets, with 70% data on the train set and 30% data on the test set, for model calibration and validation purposes. Of the subsidence training samples, approximately 84.5% belong to the <1 cm/year class, 10.5% are in the 1–5 cm/year class, and 5% are in the >5 cm/year class, creating imbalance in the dataset. Machine learning models with imbalance datasets are often biased towards the majority observation class^68^. A “balanced” class weight was assigned to prevent the model from being biased towards the majority class (<1 cm/year here). This approach calculates class weight using an inverse relation to the number of observations in a class^69^, and assigns the lowest class weight value to the most frequent class and the highest value to the least frequent class. The class weights are considered during node splitting and weighted majority voting of ensemble results to assign more penalty on misclassifying the least frequent classes (1–5 cm/year and >5 cm/year)^70^, and thus help to compensate for the imbalance in the dataset.

The hyperparameter-tuned, weight adjusted random forest model was used to generate a global map of land subsidence and a subsidence probability map (Supplementary Figs. 2 and 3). The generated land subsidence prediction was further refined with a land use filter to filter out subsidence predictions over areas where there are both low normalized irrigated area density (<0.06) and low normalized population density (<0.009). Values (unitless, ranging from 0 to 1) of normalized irrigated area density and population density where subsidence have been observed in training data are >0.1 and >0.005, respectively, but if the values are at the lower end for any one of them, the value of the other variable tends to be higher. For example, in Hefei, China, 1–5 cm/year subsidence have been observed in populated area with density ranging from 0.006–0.009 but normalized irrigated area density of >0.3. Therefore, the land use filter was only applied on areas where both of the land use variables have very low values. This land use filter removed ~7% of the 1–5 cm/year and >5 cm/year predicted subsidence pixels, mostly prediction noise generated by the model, resulting in the final global map of land subsidence (Fig. 1) induced by groundwater over-drafting. Finally, we used the subsidence map to estimate a permanent groundwater storage loss volume of ~17 km^3^/year, assuming average subsidence values of 3 and 10 cm/year for the 1−5 cm/year, >5 cm/year subsidence classes, respectively. The lower bound of permanent groundwater storage loss volume was estimated to be ~11.5 km^3^/year; assuming 2 and 7 cm/year subsidence for the 1–5 cm/year, >5 cm/year subsidence classes, respectively. The upper bound was estimated to be ~22 km^3^/year; assuming 4 and 13 cm/year subsidence for the 1–5 cm/year, >5 cm/year subsidence classes, respectively.

### Model performance

Evaluating model robustness by simply calculating the fraction of predictions that are correct can result in a biased view of model performance, particularly with an imbalanced dataset such as ours. For instance, if our model always predicts <1 cm/year of subsidence, it would be right 80% of the time, but would be ineffective. In such cases, the F1-score is an efficient metric to assess the model as it considers true positives, false negatives, and false positives in calculating accuracy. The F1-score is defined as the harmonic mean of precision and recall, where precision represents the number of true positives divided by the number of model-predicted positives, and recall represents the number of true positives divided by the sum of true positives and false negatives^69^. Performance of the model was assessed with the F1-score on the testing set for individual classes and for all classes. The F1-scores for <1 cm/year, 1–5 cm/year, and >5 cm/year were 0.96, 0.68, and 0.86, respectively. For all the classes combined, the macro F1-score (average of F1-score of individual classes) was 0.83. To avoid overfitting, the hyperparameters were optimized on the train set and the model’s performance was evaluated on the test set. Supplementary Table 5 shows F1-score for discrete classes and for the entirety.

A confusion matrix (Supplementary Fig. 13) of the test set shows that the model misclassified approximately 24% of the 1–5 cm/year class, compared to ~5.8% in the <1 cm/year and ~9.3% in the >5 cm/year classes. Despite a relatively lower F1-score, the accuracy of the 1–5 cm/year class is still quite high.

### Leave-One-Area-Out accuracy test

Performance of a random forest model is greatly influenced by the quantity, distribution, and class balance of the training dataset. Class imbalance in the model can affect individual class accuracy and overall model performance^71^. Moreover, prediction of positive class in a region depends on the number and proximity of training samples in that vicinity^72^. The random forest model developed in this study suffers from all these challenges. These challenges were minimized by assigning class weights to reduce the effect of class imbalance and by expanding the subsidence training dataset with subsidence data collected from various sources and over many regions across the globe. Despite the effort, the quantity of training samples may not be sufficient considering the global extent of the model and they may not represent all combinations of groundwater stressed regions with variety of climate, hydrology, and geology. In such cases, our model might not be able to map subsidence in small regions whose characteristics were not represented in the training dataset. Therefore, to test the model accuracy and robustness further, a Leave-One-Area-Out (LOAO) accuracy test was designed, inspired by a popular machine learning model evaluation technique called Leave-One-Out cross-validation^73^. In the “Random forests model prediction” section, it was mentioned that the original training dataset was randomly split into train and test set for model fitting and validation purposes, respectively. The random split ensured that the train set included subsidence pixels from all regions to provide the model with varying information from respective regions. In the LOAO method, the dataset was not randomly split. Instead, the model iterated *n* times, where *n* is the number of training regions, leaving one training area completely out (performed as a test set) from training on each iteration and evaluating the model performance on the excluded region. Thus, the model ran 47 times (excluding the coastal datasets during this analysis), each time excluding an area. Subsiding areas globally vary in terms of climate, hydrologic balance, and geologic formation. Removing one area from the training data may decrease the model’s prediction power over that region to some extent and sometimes entirely. Therefore, the model performance was evaluated based on subsidence probability, rather than original model subsidence prediction. The original model probability vs LOAO test probability for some regions of the world is presented in Supplementary Fig. 13. Supplementary Table 6 shows detail LOAO test results for the 47 training regions of the model, which were categorized based on criteria introduced in Supplementary Note 2. Out of the 47 regions only 10 were categorized “not satisfactory” according to the criteria. This means that the model cannot detect subsidence in these 10 regions without being explicitly trained with the deformation data of these regions. This may be due to the distinctive hydrologic, climatic, and geologic features of these regions that drive subsidence, which is not represented by any other area in the training dataset. However, the model performs satisfactorily for the rest of the 47 regions indicating robust model performance.

### Supplementary information

Supplementary Information

Peer Review File

### Source data

Source Data

## Data Availability

The hydrological, geological, elevation, and remote sensing datasets have been cited throughout the paper, listed in Supplementary Table 1, and are publicly available. Sources of the secondary subsidence datasets have been listed in Supplementary Table 3 and are publicly available. The primary subsidence datasets (from InSAR) processed by the authors are available upon request from the corresponding author. The processed training subsidence data, processed input variables, training csv file, and reference files to run the modeling scripts, along with the global subsidence and subsidence probability prediction rasters by the model, are available at this HydroShare repository^58^—10.4211/hs.dc7c5bfb3a86479b889d3b30ab0e4ef7. Data used for mapping purposes, such as global country-level shapefile and base map, are open-source datasets and have been used under appropriate license. Source data are provided with this paper.

## Code availability

The project’s modeling scripts^16^ are available at the following GitHub repository: https://github.com/mdfahimhasan/Global-Subsidence-Groundwater.
