# Supplementary material for: Global land subsidence mapping reveals widespread loss of aquifer storage capacity
Source: Nat Commun. 2023 Oct 4;14:6180. doi: 10.1038/s41467-023-41933-z (PMC10550978; doi:10.1038/s41467-023-41933-z)
Supplement: Supplementary file 1 — Supplementary Information [file 41467_2023_41933_MOESM1_ESM.pdf]

Supplementary Materials

Global Land Subsidence Mapping Reveals Widespread Loss of Aquifer Storage Capacity

Md Fahim Hasan[1], Ryan Smith[1], Sanaz Vajedian[2], Rahel Pommerenke[1], Sayantan Majumdar[3]

[1] Department of Civil and Environmental Engineering, Colorado State University, Fort Collins, CO, 80523, USA

[2] Department of Geosciences and Geological and Petroleum Engineering, Missouri University of Science and Technology, Rolla, MO, 65409, USA

[3] Division of Hydrologic Sciences, Desert Research Institute, Reno, NV, 89512, USA

Corresponding Author: Md Fahim Hasan (Fahim.Hasan@colostate.edu)

Supplementary Figures

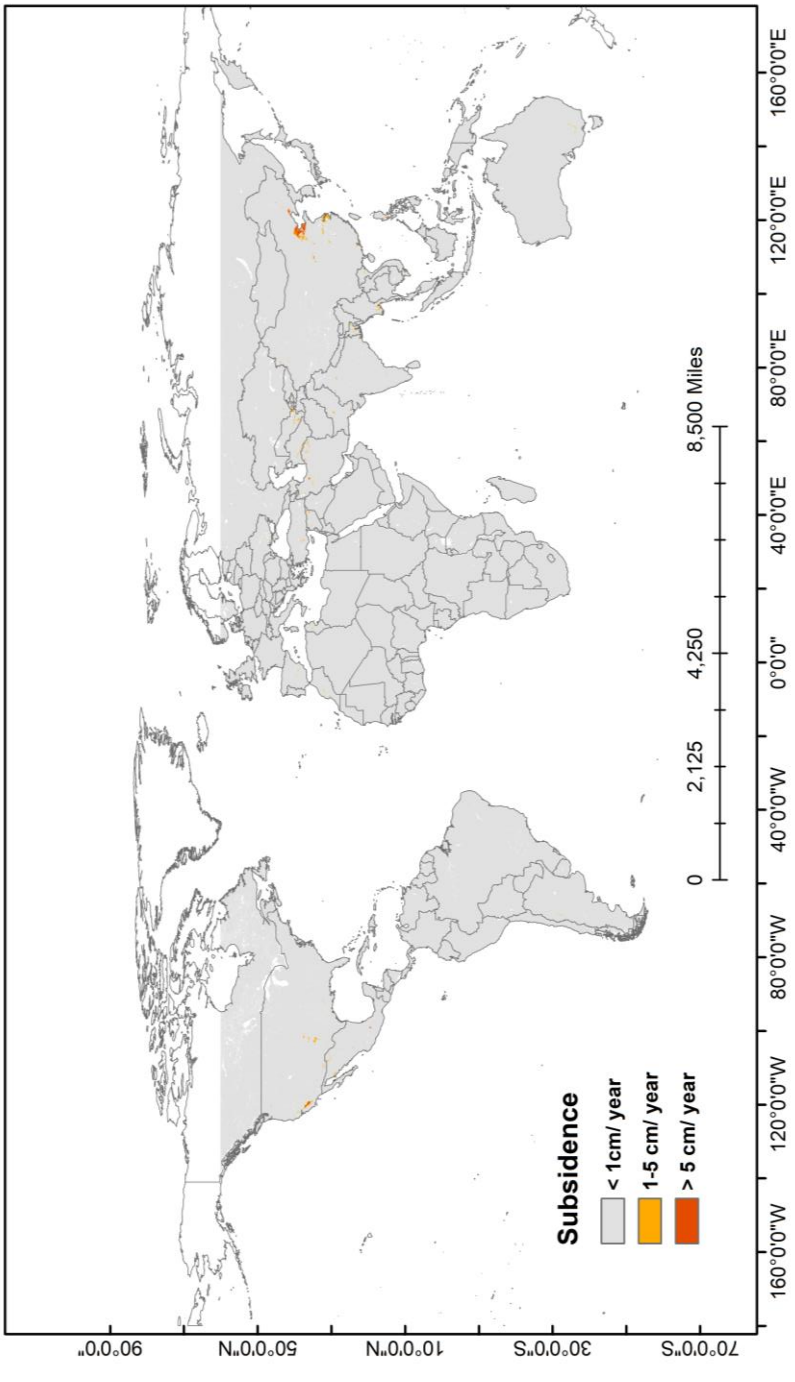

Supplementary Fig. 1: Global map of Land Subsidence at ~2 km spatial resolution. A high-quality map is available at this GitHub link. Source data are provided as a Source Data file.

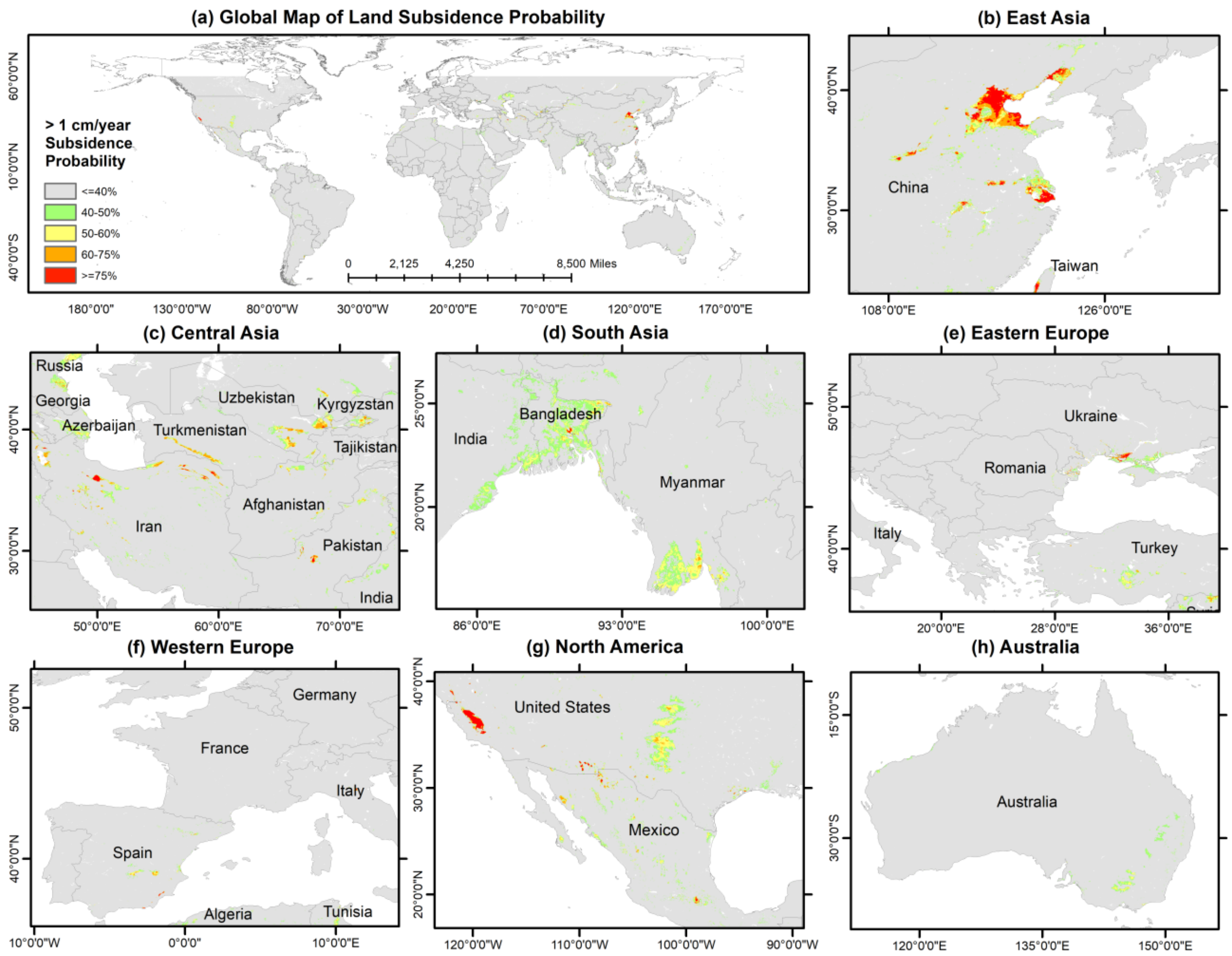

Supplementary Fig. 2: Probability of subsidence of magnitude >1 cm/year for regions with high subsidence probability. (a) shows the global map of subsidence probability of >1 cm/year magnitude. Warmer color signifies a higher probability of subsiding more than 1 cm/year and vice versa. The model shows significant >1 cm/year subsidence probability across the globe that covers regions in (b) East Asia, (c) Central Asia, (d) South Asia, (e) Eastern Europe, (f) Western Europe, (g) North America, and (h) Australia. A high-quality map is available at this GitHub link. Source data are provided as a Source Data file.

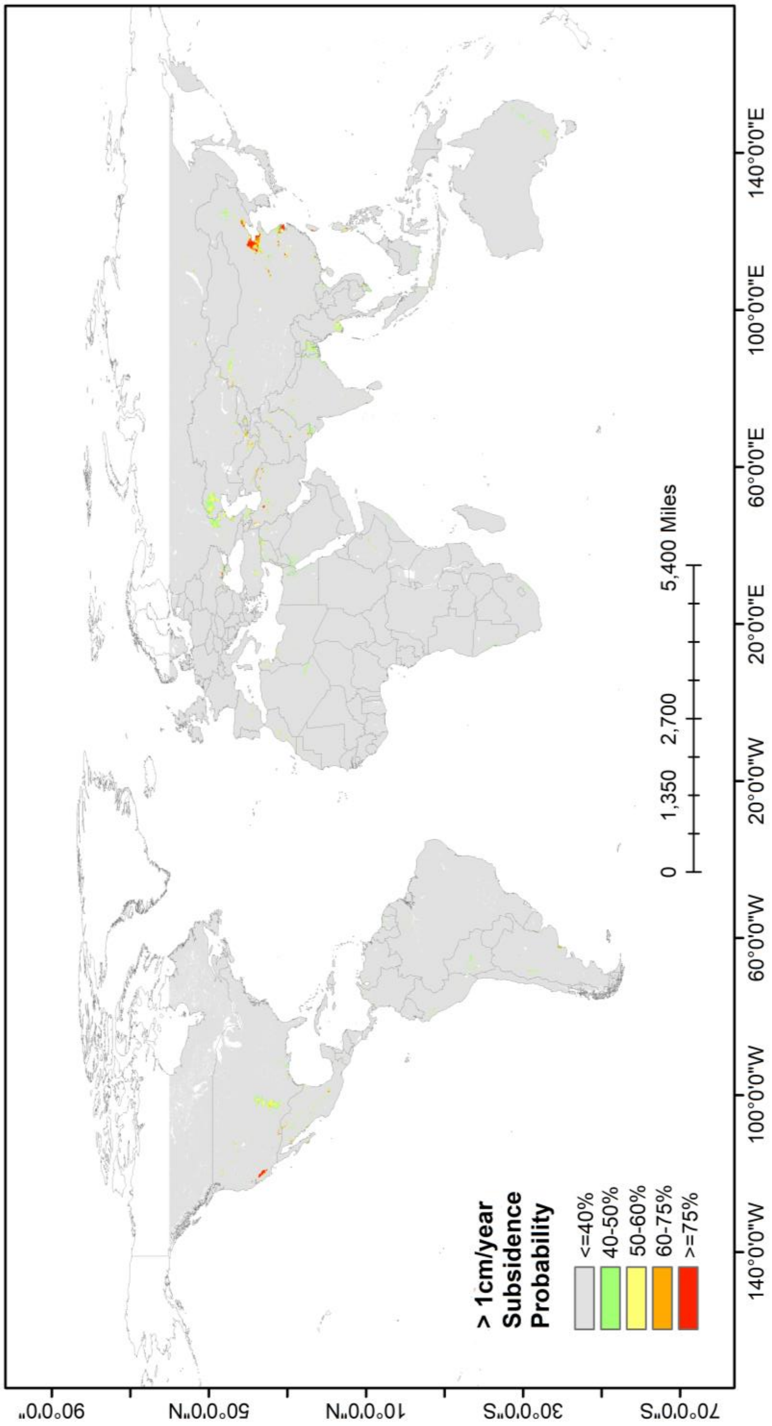

Supplementary Fig. 3: Global Map of Probability of >1 cm/year Subsidence. Warmer color signifies a higher probability of subsiding more than 1 cm/year. A high-quality map is available at this GitHub link. Source data are provided as a Source Data file.

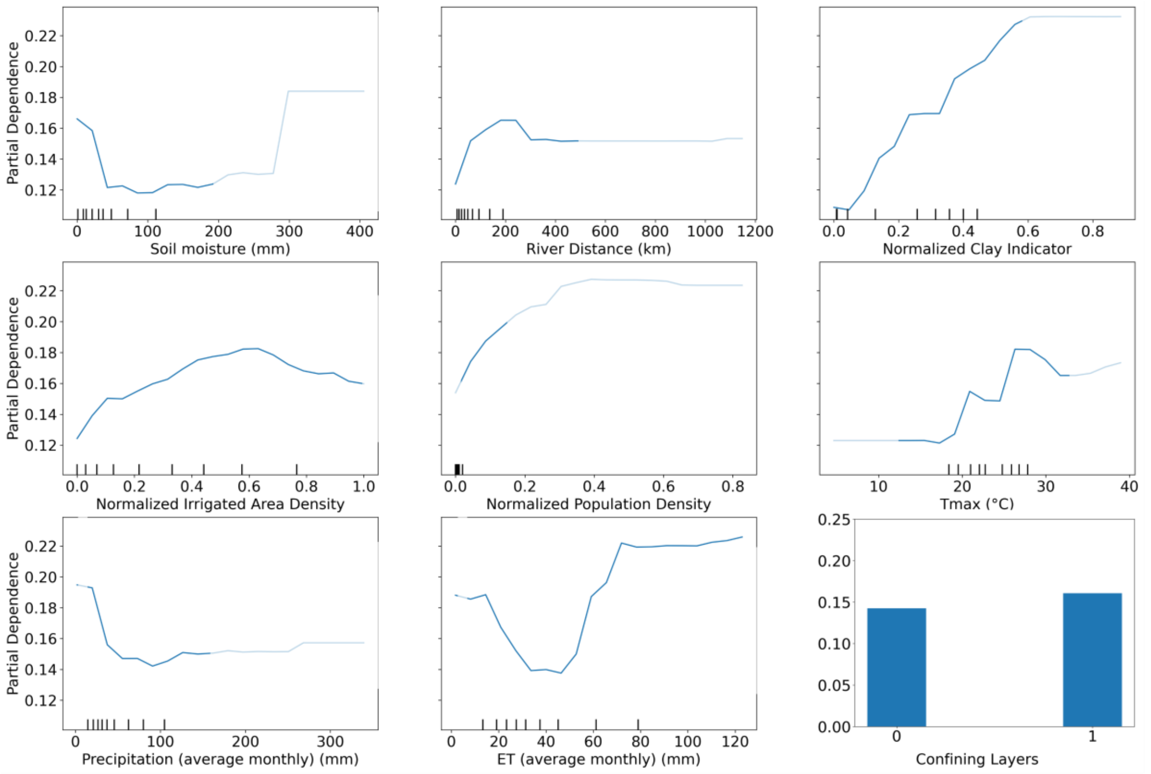
Partial Dependence
0.12
0.14
0.16
0.18
0.20
0.22
0
100
200
300
400
Soil moisture (mm)
0
200
400
600
800
1000
1200
River Distance (km)
0.0
0.2
0.4
0.6
0.8
Normalized Clay Indicator
0.0
0.2
0.4
0.6
0.8
1.0
Normalized Irrigated Area Density
Normalized Population Density
10
20
30
40
Tmax (°C)
Precipitation (average monthly) (mm)
0
20
40
60
80
100
120
ET (average monthly) (mm)
0.00
0.05
0.10
0.15
0.20
0.25
0
1
Confining Layers

(a)

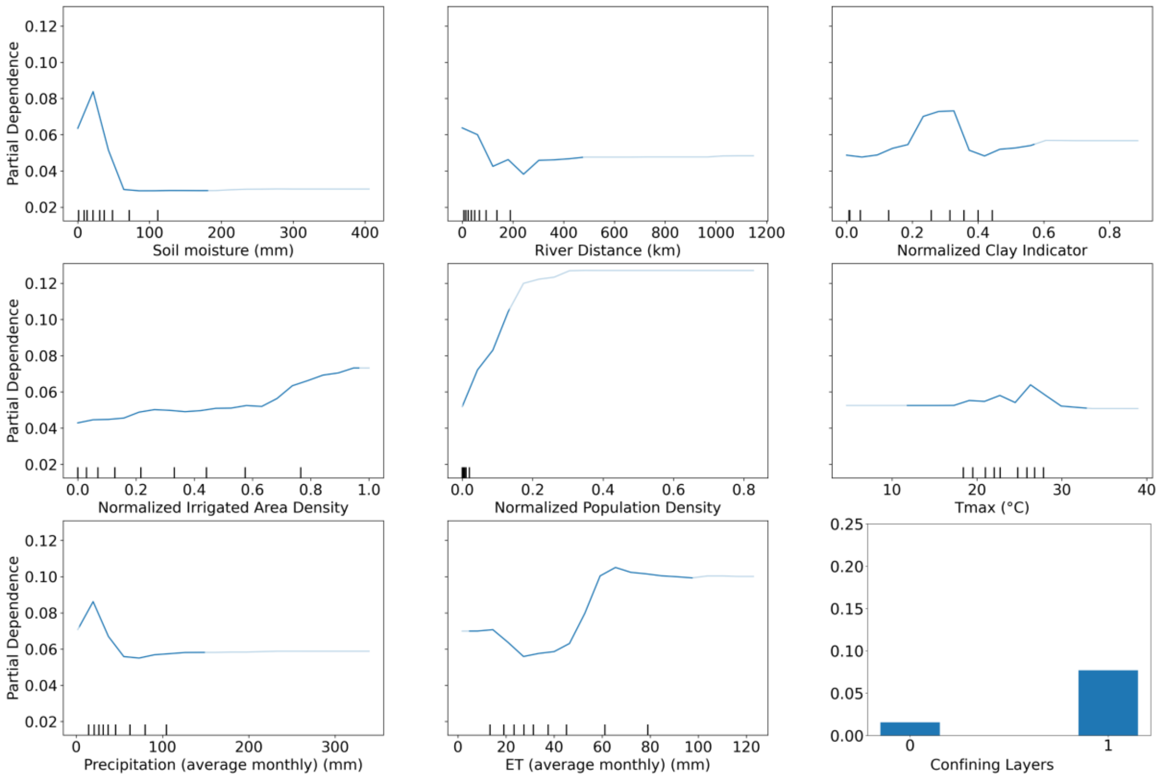
Partial Dependence
0.02
0.04
0.06
0.08
0.10
0.12
0
100
200
300
400
Soil moisture (mm)
0
200
400
600
800
1000
1200
River Distance (km)
0.0
0.2
0.4
0.6
0.8
Normalized Clay Indicator
0.0
0.2
0.4
0.6
0.8
1.0
Normalized Irrigated Area Density
Normalized Population Density
10
20
30
40
Tmax (°C)
Precipitation (average monthly) (mm)
0
20
40
60
80
100
120
ET (average monthly) (mm)
0.00
0.05
0.10
0.15
0.20
0.25
0
1
Confining Layers

(b)

Supplementary Fig. 4: The partial dependence plot (PDP) for the (a) 1-5 cm/year class, (b) > 5 cm/year class. Here, Tmax and ET stand for maximum temperature and evapotranspiration, respectively. The x-axis of the plots consists of variable values and the y-axis represents how the subsidence prediction varies dependent on a variable while accounting for the average influence off all other predictor variables in the model[1]. The responses of variables in predicting subsidence are more interpretable within the 1st to 99th percentiles as this range covers most of data (dark blue lines in the plots). For some variables, such as soil moisture, river distance, normalized clay indicator, normalized irrigated area density, the 1st percentile value is 0, so, there is no shade in that side of the plot. In contrast to the other variables, the confining layer only has 2 values (1 for presence of confining units, 0 for no confining units). The response of the 'Confining Layer' variable in the model is more explainable if PDP plots for this predictor for all classes (<1 cm/year, 1-5 cm/year, >5cm/year) are analyzed together (Supplementary Fig. 5).

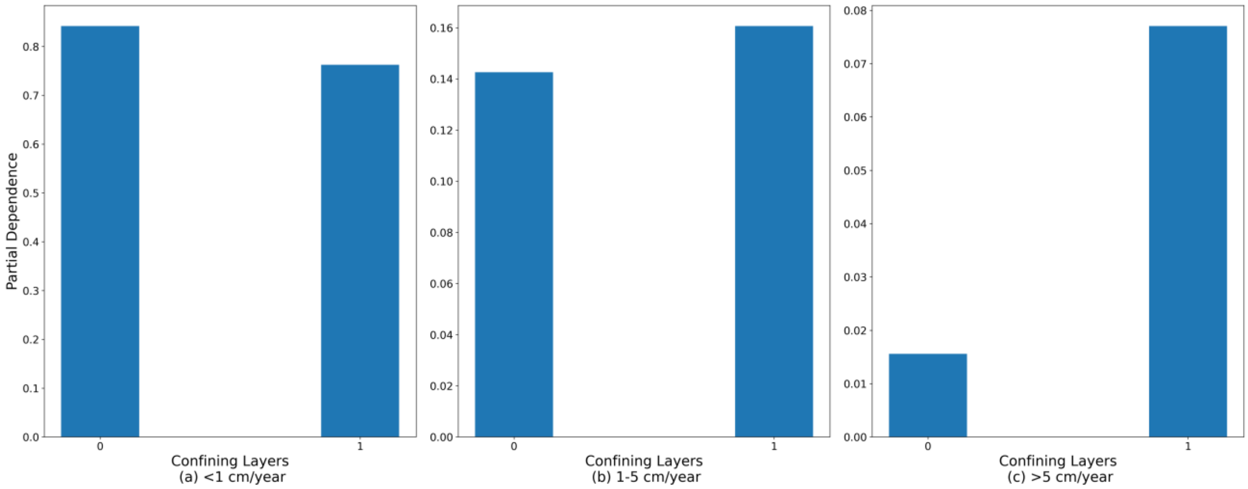

Supplementary Fig. 5: Partial Dependence Plots in variable ‘Confining Layers’ for all model classes. In contrast to other variables, the confining layer only has 2 values (1 for presence of confining units, 0 for no confining units). For the <1 cm/year class, the prediction probability is high for no confining layer (value 0), signifying that- areas with no confining units tend to be less vulnerable to high subsidence. For the other classes, the probability of subsidence increases with the existence of confining units (value 1).

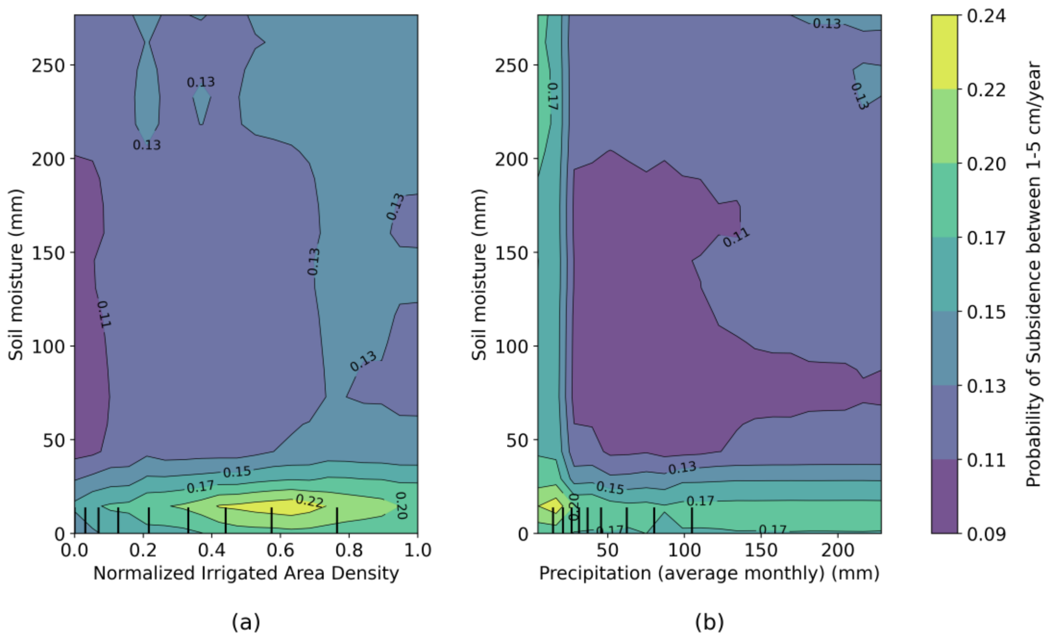

Supplementary Fig. 6: Partial dependence plots of (a) Soil Moisture and Normalized Irrigated Area Density, (b) Soil Moisture and Precipitation in predicting 1-5 cm/year subsidence. Warmer color indicates higher subsidence probability. The values in both axes have been plotted between 1st to 99th percentile as the model's response (in predicting subsidence) to the variables is more evident within this percentile range. The plots represent how soil moisture plays an important role in subsidence prediction despite not being a direct driver of subsidence. A combination of low soil moisture and high irrigation density leads to higher probability of subsidence (Fig. 6(a)). Fig 6(b) shows that the model predicts higher probability of subsidence in combination of low precipitation and low soil moisture. Note that the subsidence probability contour in Fig 6(b) varies a little from the similar plot in Fig. 2 of the main text, though both plots were generated using the same trained random forest model. This is due to the fact that while constructing Fig. 6 (supplementary materials) and Fig. 2 (main text), the combinations of variables were different, but both plots indicate a similar response to subsidence prediction. The values of precipitation are average monthly precipitation.

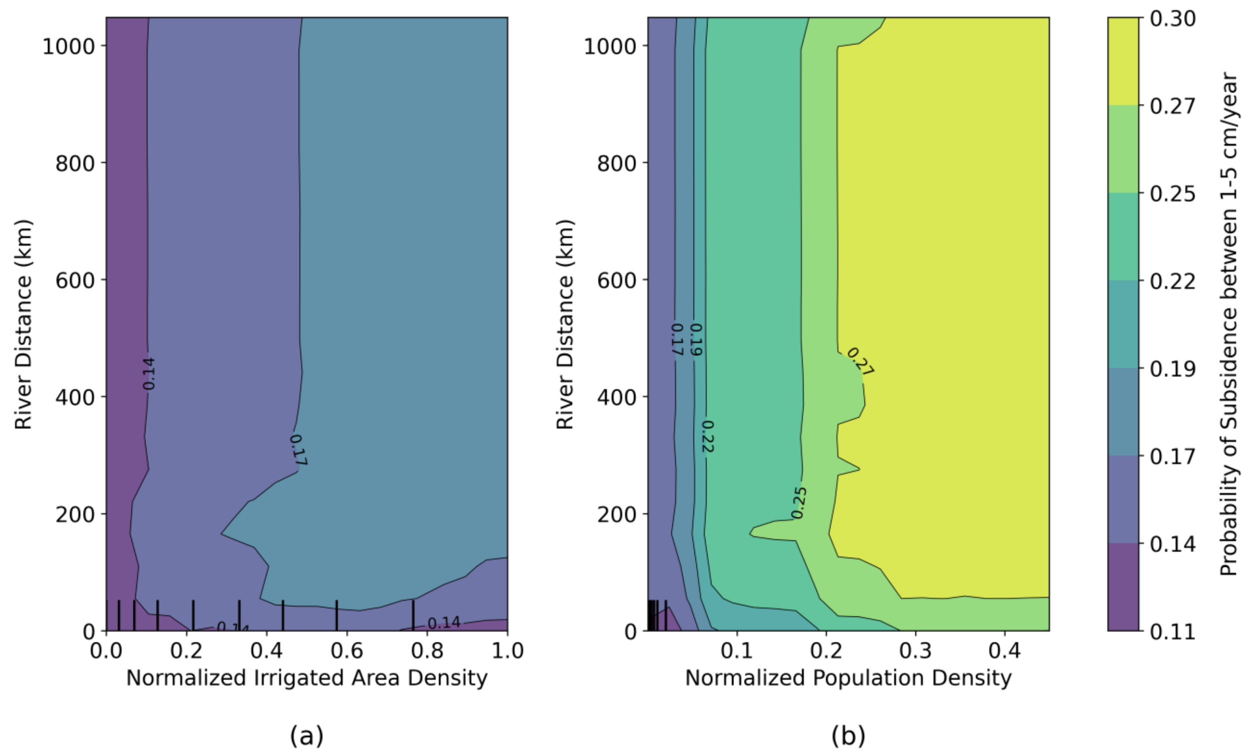

Supplementary Fig. 7: Partial dependence plots of (a) River Distance and Normalized Irrigated Area Density and (b) River Distance and Population Density in predicting 1-5 cm/year subsidence. Warmer color indicates higher subsidence probability. The values in both axes have been plotted between 1st to 99th percentile as the model's response (in predicting subsidence) to the variables is more evident within this percentile range. The plots represent how river distance plays an important role in subsidence prediction despite not being a direct driver of subsidence. Groundwater is the main source of irrigation water and water supply in agricultural lands and populated areas distant from surface water sources. In such regions, excessive groundwater withdrawal can lead to subsidence. The plots show a higher probability of subsidence in areas with high irrigation and population density if they are also distant from rivers.

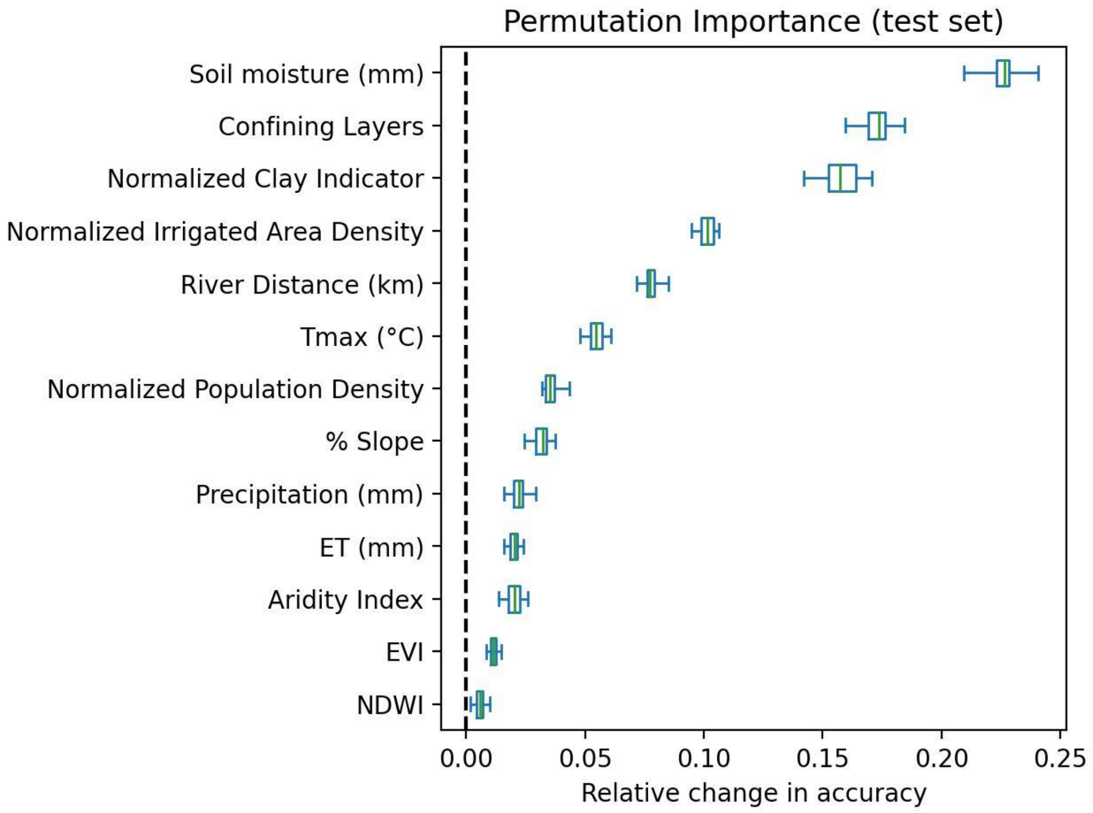

Supplementary Fig. 8: Variable importance plot for the model predictors in the random forests model based on Permutation importance. From top to bottom the relative importance decreases. Soil moisture, confining layers, normalized clay indicator, and normalized irrigated area density are the top four predictors. Here, permutation importance is evaluated on the test dataset. Permutation importance runs the model multiple iterations and shuffles a input variable's value in each iteration to evaluate relative change in model performance[2]. For variables that cause the most change in relative model accuracy (macro-F1 in this case) due to the permutation are considered the most important predictors.

Here, Tmax: Maximum Temperature; ET: Evapotranspiration; EVI: Enhanced Vegetation Index; NDWI: Normalized Difference Water Index

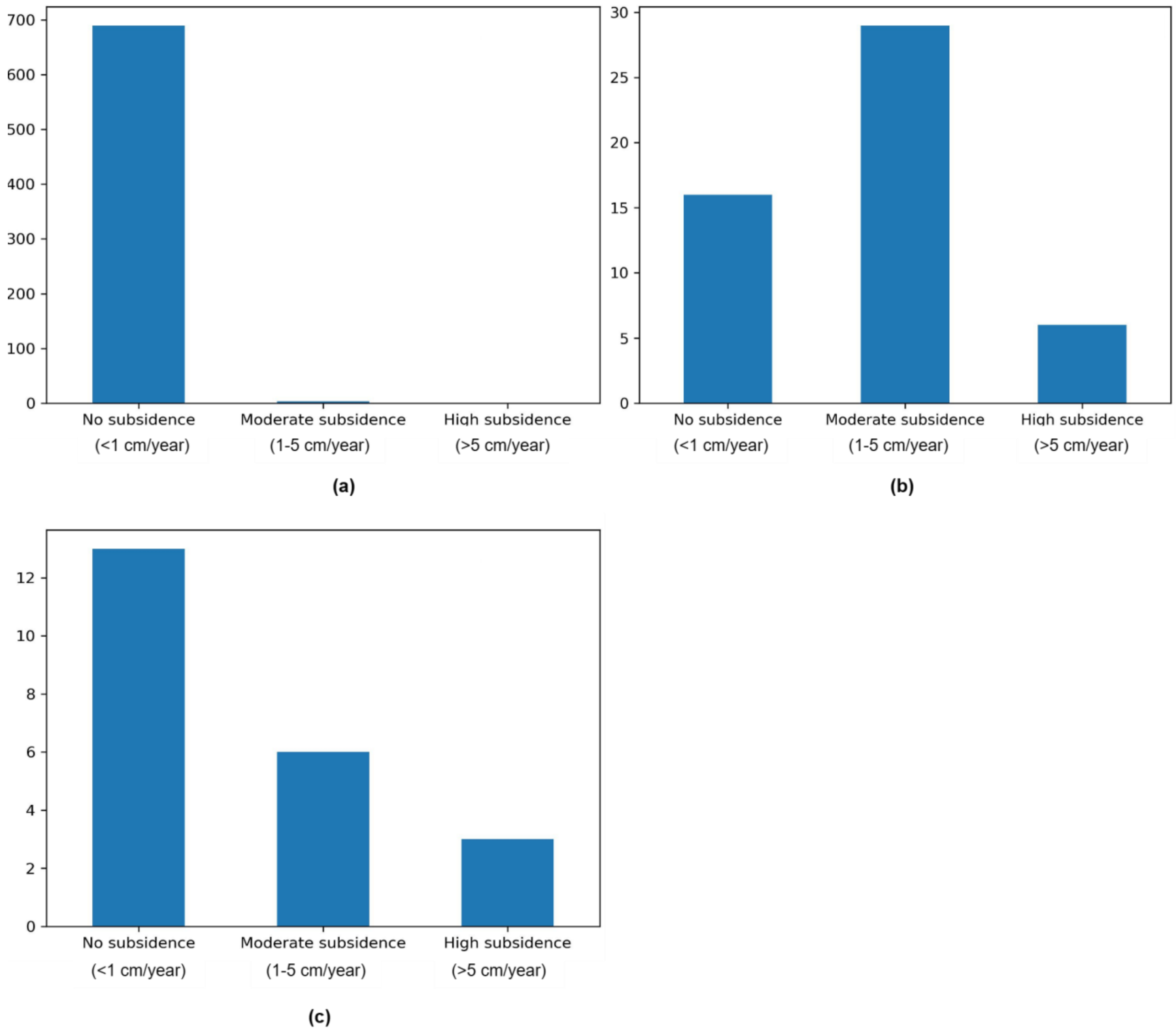

Supplementary Fig. 9: Results from soil moisture sensitivity analysis test for a combination of (a) low soil moisture – no confining layers – low normalized clay indicator – low normalized irrigated area density, (b) low soil moisture – presence of confining layers – high normalized clay indicator – high normalized irrigated area density, and (c) high soil moisture – presence of confining layers – high normalized clay indicator – high normalized irrigated area density. The results have been discussed in detail in Supplementary Section 2. The selection criteria of the variables are presented in Supplementary Table 7.

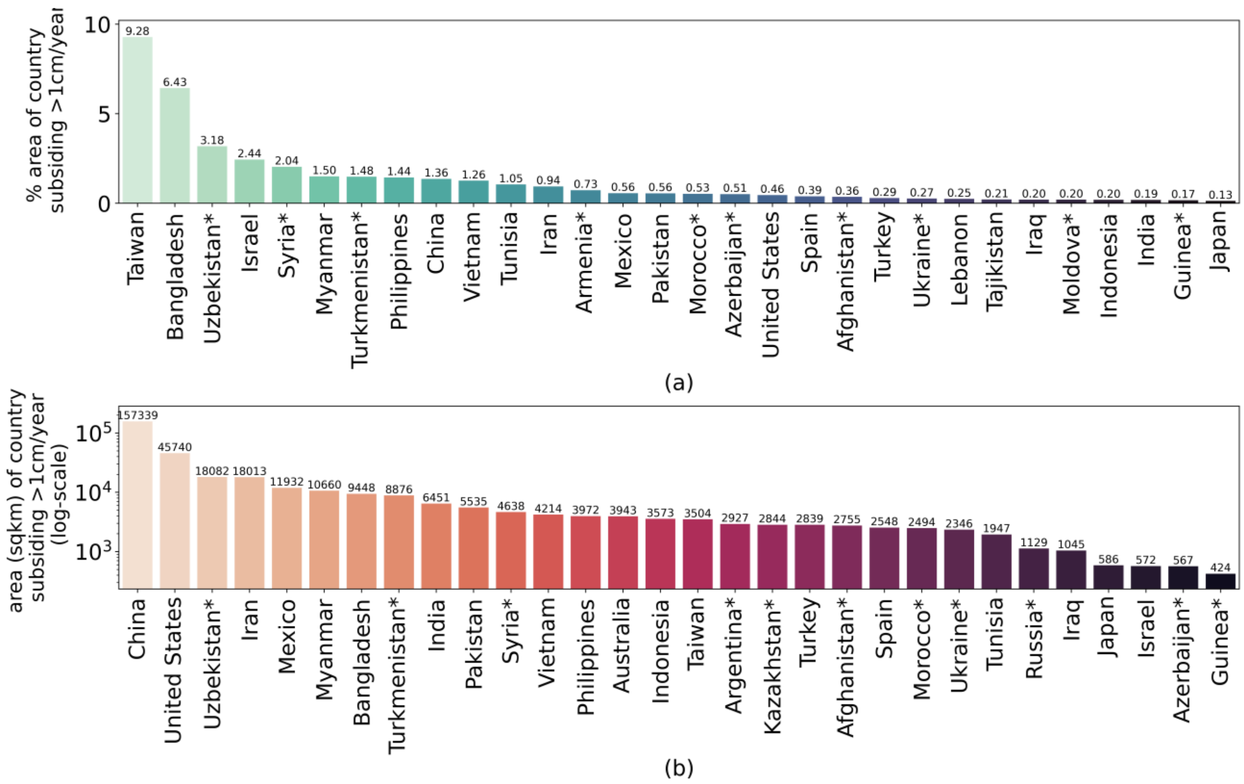

Supplementary Fig. 10: Country statistics of subsidence prediction of magnitude >1 cm/year. (a) shows countries with the highest percentage of subsidence with respect to their land area, (b) shows countries with the highest area of subsidence predicted by our model. Countries with asterisks are where no previously published land subsidence studies due to groundwater withdrawal were available. Source data are provided as a Source Data file.

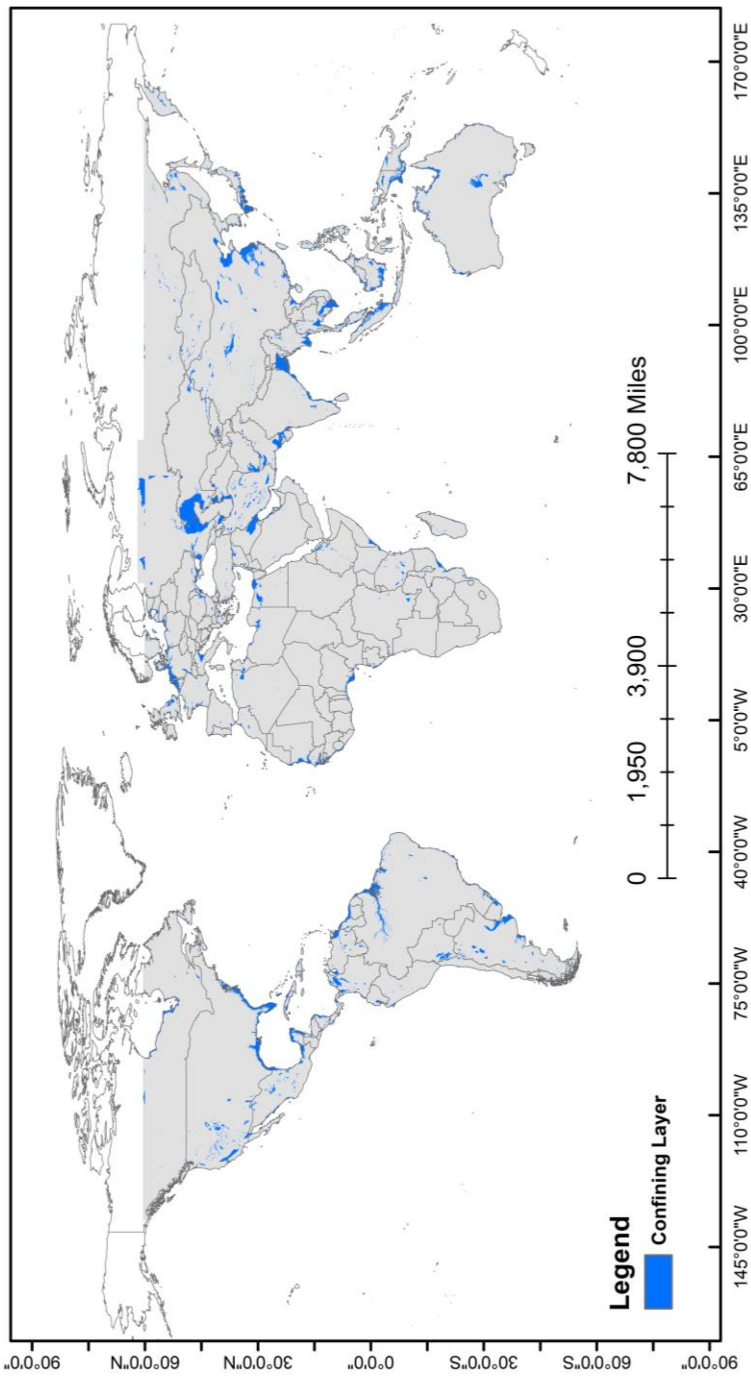

Supplementary Fig. 11: Likely presence of confining layers. These regions are likely to have extensive oceanic or lacustrine depositional environments containing fine grained sediments that might undergo subsidence with groundwater pumping. Source data are provided as a Source Data file.

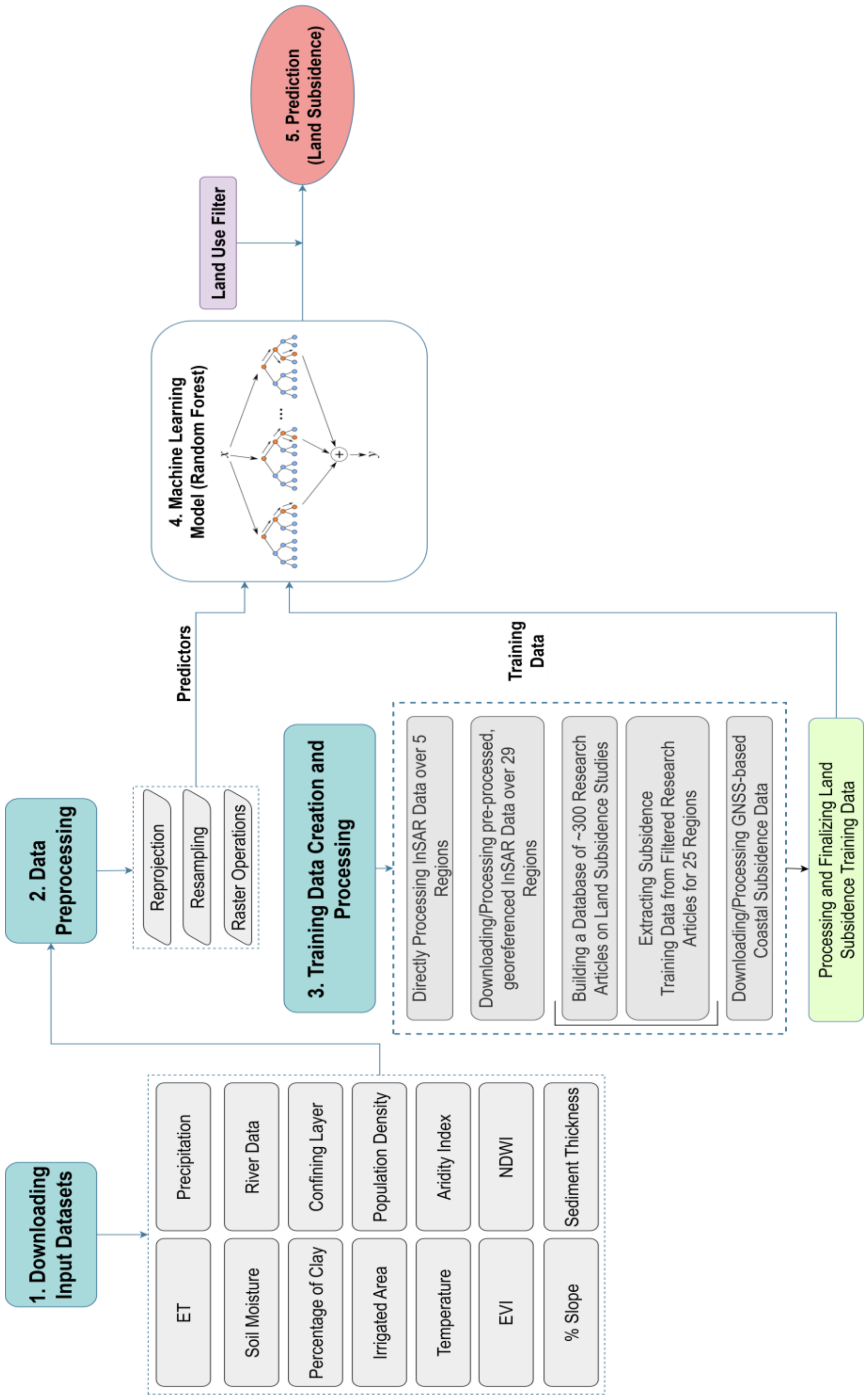

Supplementary Fig. 12: Framework of the machine learning model including data preprocessing, training data creating steps. Random forests algorithm has been incorporated in Step 4 of the model. The random forests figure was downloaded from the IU Digital Science Center[3].

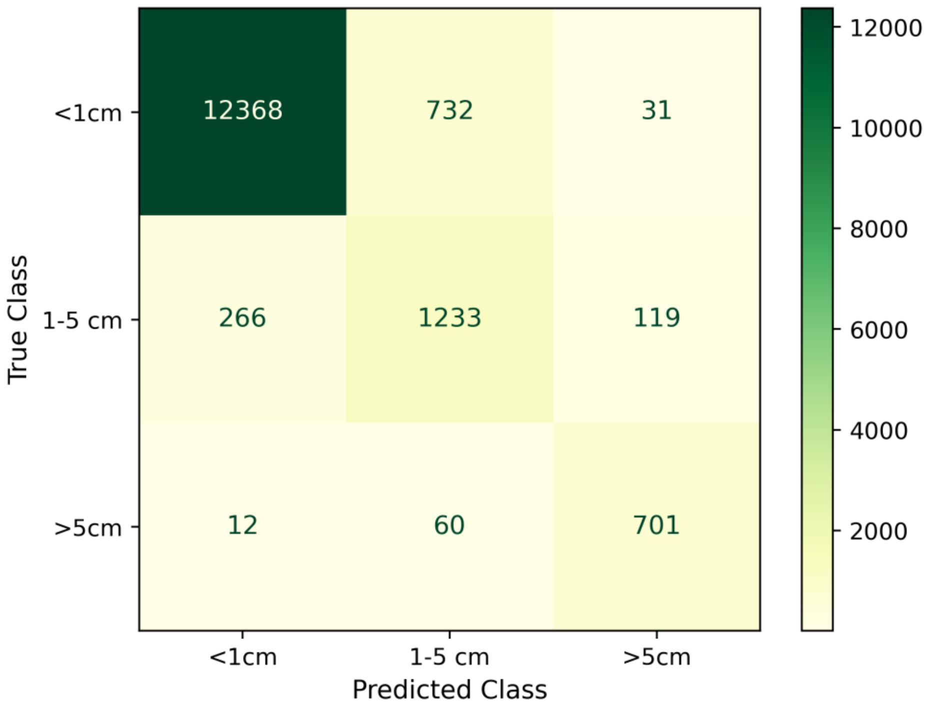

Supplementary Fig. 13: Confusion matrix of test set. 94.2% and 90.7% observations of <1cm/year and >5 cm/year classes, respectively, were classified accurately. For 1-5 cm/year class, the accuracy is approximately 76.2%. Source data are provided as a Source Data file.

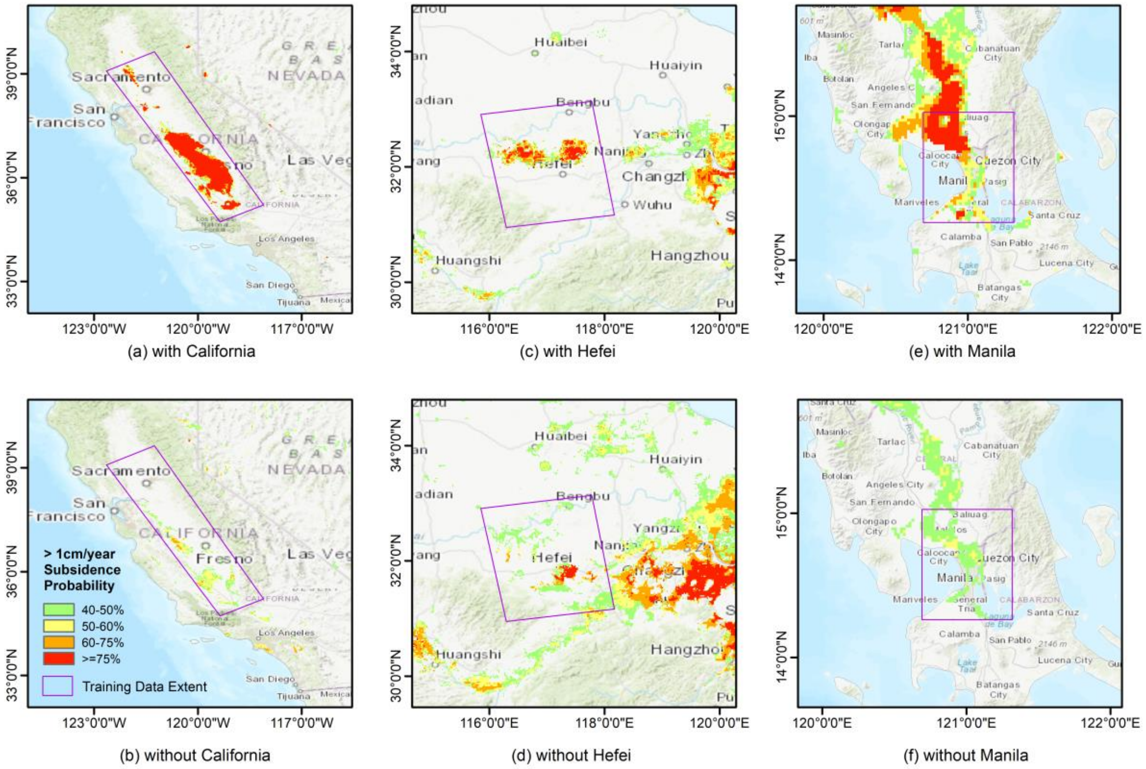

Supplementary Fig. 14: Leave-One-Area-Out subsidence probability with respect to original subsidence probability. (a-b), (c-d), (e-f) map shows subsidence probability with and without California, USA; Hefei, China; and Manila, Philippines regions' training data. Source data are provided as a Source Data file.

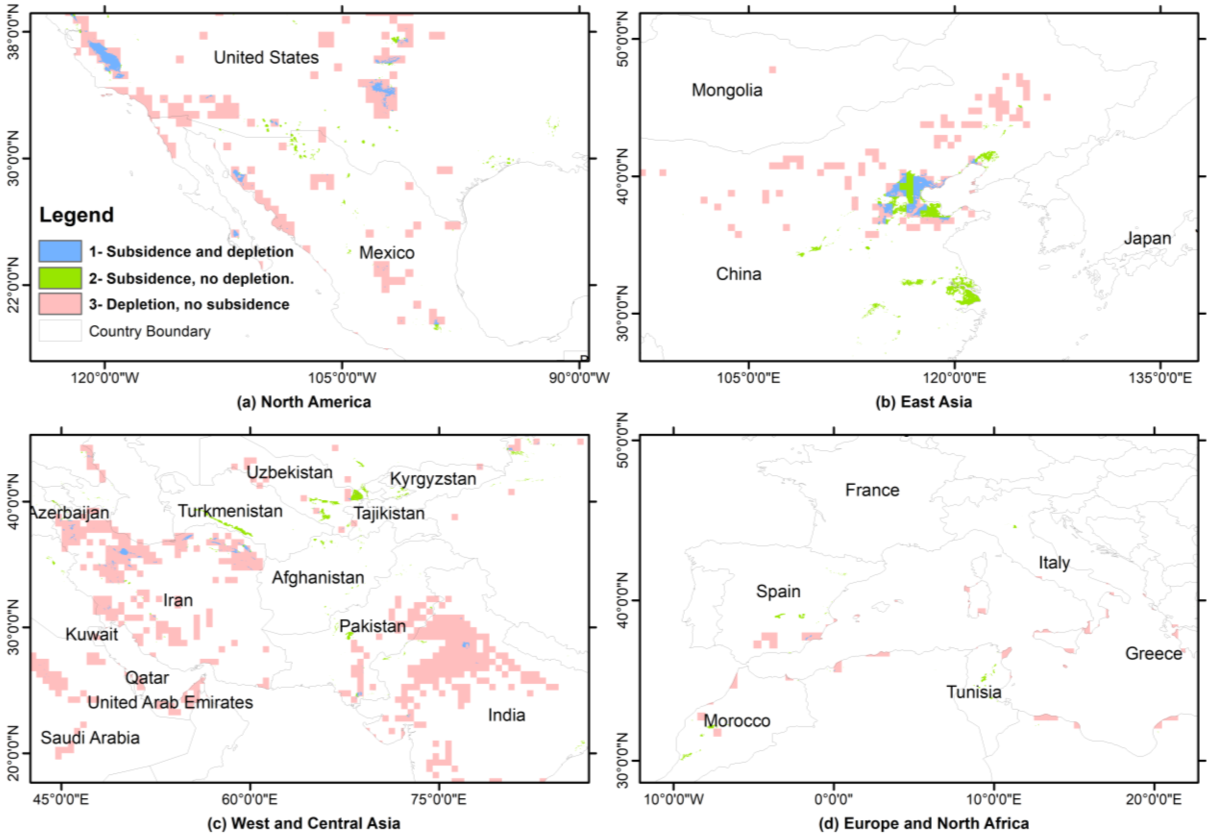

Supplementary Fig. 15: Comparison of model result with groundwater depletion estimate by Wada et al.[4] in (a) North America, (b) East Asia, (c) West and Central Asia, and (d) Europe and North Africa. Here, 1- Subsidence and depletion (both study results match); 2- our study predicts subsidence while Wada et al.[4] does not show depletion; 3- Wada et al.[4] estimates large groundwater decline while our study does not show subsidence. Source data are provided as a Source Data file.

Supplementary Tables

Supplementary Table 1: List of input datasets used in the model.

Input Variable	Spatial resolution	Temporal Aggregation method	Source
Soil moisture	~4 km	mean	Abatzoglou et al.[5]
Precipitation	~4 km	monthly mean	Abatzoglou et al.[5]
Evapotranspiration	~4 km	monthly mean	Abatzoglou et al.[5]
Irrigated Area	~1 km	stationary	Meier et al.[6]
Population Density	~1 km	mean	CIESIN[7]
Temperature	~4 km	monthly median	Abatzoglou et al.[5]
Percentage of Clay	250 m	stationary	Hengl[8]
Sediment Thickness	~1 km	stationary	Pelletier et al.[9]
Major River	Vector data	stationary	GRDC[10]
EVI	250 m	mean	Didan[11]
NDWI	500 m	mean	Vermote[12]
Aridity Index	~1 km	stationary	Trabucco & Zomer[13]
DEM	30 m	stationary	Farr et al.[14]

Supplementary Table 2: Comparison of the presence of a confining layer as mapped by our method with aquifer reports from the USGS (2000)[15].

Name	% area with confining layer present	Presence of extensive confining layer from the literature	Chapter from USGS (2000)[15]
California Coastal Basin aquifers	15	Yes	B
Central Valley aquifer system	73	Yes	B
Coastal lowlands aquifer system	40	Yes	E
Columbia Plateau basin-fill aquifers	4	Yes	H
High Plains aquifer	0	No	D
Mississippi Embayment aquifer	0	Yes	F
Mississippi River Valley alluvial aquifer	4	No	F
Northern Atlantic Coastal Plain aquifer system	56	Yes	M
Northern Rocky Mountains Intermontane Basins aquifer system	6	Yes	H
Pecos River Basin alluvial aquifer	0	No	E
Rio Grande aquifer system	1	Yes	C
Seymour aquifer	0	No	E
Snake River Plain basin-fill aquifers	20	Yes	H

Name	% area with confining layer present	Presence of extensive confining layer from the literature	Chapter from USGS (2000)[15]
Southeastern Coastal Plain aquifer system	1	Yes	G
Surficial aquifer system	51	Yes	G
Texas coastal uplands aquifer system	0	Yes	E

Supplementary Table 3: Source of training InSAR data. (1-5) were obtained by processing InSAR by the authors. (6-34) were pre-processed, georeferenced data collected from public agencies of the United States and European Ground Motion Service (EGMS). (35-59) were georeferenced and classified from published research articles. 60 is globally available Global Navigation Satellite System (GNSS)-based coastal subsidence data.

**Processed by the authors**				
**No.**	**Country**	**Region**	**Temporal Window**	**Sources**
1	China	Hebei	2015-2019	authors
2	China	Hefei	2015-2019	
3	Iran	Qazvin	2014-2021	
4	USA	San Luis Valley, Colorado	2014-2021	
5	Pakistan	Quetta	2017-2021	
**Georeferenced, pre-processed subsidence data**				
6	USA	California	2015-2019	California Department of Water Resources
7	USA	Arizona	2010-2019	Arizona Department of Water Resources
8	England	London	2015-2021	EGMS
9	Denmark	Tinglev	2015-2021	EGMS
10	France	Bordeaux	2015-2021	EGMS
11	Greece	Alexandreia & Palamas	2015-2021	EGMS
12	Greece	Patras & Katochi	2015-2021	EGMS
13	Hungary	Szeged	2015-2021	EGMS
14	Romania	Timisoara	2015-2021	EGMS
15	Italy	Cerignola Campagna	2015-2021	EGMS
16	Italy	Mazzafarro	2015-2021	EGMS
17	Italy	Po Delta	2015-2021	EGMS
18	Italy	Rosarno	2015-2021	EGMS
19	Italy	Salerno	2015-2021	EGMS
20	Italy	Schiavonea	2015-2021	EGMS
21	Netherlands	Limburg	2015-2021	EGMS

22	Spain	Murcia	2015-2021	EGMS
**Secondary Sources (georeferenced from research articles)**				
23	Australia	Perth	2015-2018	Castellazzi & Schmid[16]
24	Bangladesh	GBM Delta	2007-2011	Higgins et al.[17]
25	China	Beijing	2003-2010	Chen et al.[18]
26	China	Yellow River	2007-2011	Higgins et al.[19]
27	China	Shanghai	2007-2010	Dong et al.[20]
28	China	Wuhan	2015-2016	Zhou et al.[21]
29	China	Xian	2005-2012	Qu et al.[22]
30	China	Tianjin	2007-2010	Luo et al.[23]
31	Egypt	Nile Delta	2004-2010	Gebremichael et al.[24]
32	Indonesia	Multiple Locations	2006-2009	Chaussard et al.[25]
33	Indonesia	Bandung	2007-2011	Ge et al.[26]
34	Iran	Marand Plain	2015	Andaryani et al.[27]
35	Iran	Tehran	2015-2017	Haghighi & Motagh [28]
36	Iran	Mashhad	2014-2017	Khorrami et al.[29]
37	Iraq	Tigris Euphrates Basin	2015-2018	Rateb & Kuo[30]
38	India	Delhi	2016-2019	Garg et al.[31]
39	Mexico	Mexico City	2007-2011	Chaussard et al.[32]
40	Nigeria	Lagos	2015-2018	Cian et al.[33]
41	Philippines	Manila	2014-2017	Zoysa et al.[34]
42	Turkey	Karapinar	2014-2018	Orhan et al.[35]
43	Turkey	Bursa	2014-2017	Aslan et al.[36]
44	Taiwan	Yunlin	2006-2007	Hung et al.[37]
45	USA	Houston	2007-2011	Miller & Shirzaei[38]
46	Vietnam	Ho Chi Minh	2006-2010	Minh et al.[39]
47	Vietnam	Hanoi	2015-2018	Nguyen et al.[40]
48	Coastal	Global	2006-2019	Shirzaei et al.[41]

Supplementary Table 4: Selected hyperparameters for model tuning. Five (5) key hyperparameters of the random forest model were tuned using a random search 10-fold cross-validation approach for achieving the optimal model performance. The ranges of values for each hyperparameter were chosen based on literature review of scientific articles and machine learning community's best practices. The random search 10-fold cross-validation approach was chosen for computationally efficient optimization. A total of 80 combinations of parameters (totaling to 800 total fits using 10-fold cross-validation) were fitted to the model to find the best set of parameters.

**Hyperparameters**	**Represents**	**List of values chosen for tuning**	**Fitted value**
n_estimators	number of trees	100, 200, 300, 400, 500	300
max_depth	maximum depth of a tree	8, 12, 13, 14	14
max_features	maximum number of features used in node split	6, 7, 9, 10	7
min_samples_split	minimum number of samples for node splitting	6, 7, 8, 10	7
min_samples_leaf	minimum number of samples for deciding leaf node	5^{-4}, 1^{-5}, 1^{-3}, 6, 12, 20, 25	1^{-5}

Supplementary Table 5: Performance score on test set of the model for individual classes and all classes combined. The F1-score for the individual classes and for all the classes combined are satisfactory. The difference between score values for train and test sets indicate minimal over-fitting; however, the F1-score for the 1-5 cm/year class is not as high as the other two classes. The <1 cm/year and >5 cm/year classes are the majority and minority classes, respectively, with almost 85.5% observations in the majority class and approximately 4.5% observation in the minority class. The 1-5 cm/year class has boundaries with both the majority and minority subsidence classes, which affects the ability of the model to classify the 1-5 cm/year class to some extent.

F1-score for model classes				
	**<1 cm/year**	**1-5 cm/year**	**>5 cm/year**	**Macro F1-score**
Train Set	0.98	0.83	0.95	0.92
Test Set	0.96	0.68	0.86	0.83

Supplementary Table 6: Results of Leave-One-Area-Out (LOAO) test. Thirty-four (34) regions show ‘satisfactory’ and three (03) show ‘acceptable’ accuracy. Result was ‘not satisfactory for ten (10) regions. The highlighted regions are where training data only consisted of <1 cm/year observations. The accuracy assessment categories are described in Supplementary Note 2.

**No.**	**Country**	**Regions**	**Accuracy category**	**% Pixels with > 40% probability of subsidence**
1	Bangladesh	Ganges Brahmaputra Delta	satisfactory	50.3
2	China	Beijing	satisfactory	29.9
3	China	Hebei	satisfactory	74.3
4	China	Hefei	satisfactory	12.0
5	China	Tianjin	satisfactory	70.5
6	China	Xian	satisfactory	38.2
7	China	Yellow River Delta	satisfactory	46.3
8	Greece	Alexandreia &Palamas	satisfactory	0.91
9	Indonesia	Bandung	satisfactory	20.7
10	Indonesia	Semarang	satisfactory	0.1
11	Iran	Mashhad	satisfactory	23.8
12	Italy	Cerignola Campagna	satisfactory	3.0
13	Philippines	Manila	satisfactory	14.9
14	Spain	Murcia	satisfactory	3.5
15	Turkey	Karapinar	satisfactory	13.1
16	Vietnam	Hanoi	satisfactory	79.9
17	Vietnam	HoChiMinh	satisfactory	52.1
18	**Australia**	**Perth**	satisfactory	0.0
19	**United States**	**Colorado**	satisfactory	5.2
20	**Egypt**	**Nile Delta**	satisfactory	2.7
21	**England**	**London**	satisfactory	0.0
22	**France**	**Bordeaux**	satisfactory	0.0

No.	Country	Regions	Accuracy category	% Pixels with > 40% probability of subsidence
23	**Greece**	**Patras & Katochi**	satisfactory	0.0
24	**Hungary**	**Szeged**	satisfactory	0.0
25	**Iraq**	**Tigris Euphrates Basin**	satisfactory	22.3
26	**Italy**	**Mazzafarro**	satisfactory	0.0
27	**Italy**	**Rosarno**	satisfactory	0.0
28	**Italy**	**Salerno**	satisfactory	0.0
29	**Italy**	**Schiavonea**	satisfactory	0.0
30	**Netherlands**	**Limburg**	satisfactory	0.0
31	**Nigeria**	**Lagos**	satisfactory	1.4
32	**United States**	**Huston**	satisfactory	0.0
33	**Denmark**	**Tinglev**	**Satisfactory**	**0.0**
34	**Romania**	**Timisoara**	**satisfactory**	**0.0**
35	United States	California	acceptable	2.1
36	Iran	Marand Plain	acceptable	18.5
37	Mexico	Mexico City	acceptable	6.0
38	United States	Arizona	not satisfactory	0.1
39	China	Shanghai	not satisfactory	0.0
40	China	Wuhan	not satisfactory	0.0
41	India	Delhi	not satisfactory	0.0
42	Iran	Qazvin	not satisfactory	0.2
43	Iran	Tehran	not satisfactory	0.0
44	Italy	Po Delta	not satisfactory	0.0
45	Pakistan	Quetta	not satisfactory	0.0
46	Taiwan	Yunlin	not satisfactory	0.0
47	Turkey	Bursa	not satisfactory	0.0

Supplementary Table 7: Variable selection criteria for soil moisture sensitivity analysis test. These criteria were applied on the validation dataset to create three datasets representing three scenarios (detail discussed in Supplementary Section 2). The trained model was tested on each of these scenarios to see how the model performs in different combinations of soil moisture, confining layers, normalized clay indicator, and normalized irrigated area density (hereby denoting as ‘Variables of Interest’ (VOI)). Note that the other nine (09) variables were not explicitly chosen, rather selected based on the selection criteria of the VOI.

Scenario	Soil moisture (mm)	Confining Layer*	Normalized Clay Indicator	Normalized Irrigated Area Density
1	<50	0	<0.1	0
2	<50	1	>0.5	>0.5
3	100-300	1	>0.5	>0.5

* Confining layers is a categorical variable where 0 represents no confining layers and 1 represents presence of confining layers.

Supplementary Notes

Supplementary Note 1: Mechanism of Land Subsidence

Land subsidence is the downward vertical deformation of the ground surface. For the purpose of this study, only subsidence caused by groundwater extraction was considered. Subsidence is induced when groundwater withdrawal causes the pore pressure in the aquifer to decrease, resulting in an increase in effective stress and subsequent compaction in aquifer sediments[42,43]. Weak sediments, such as clay, compact more than stronger sediments like sand, gravel, or consolidated rock. Thus, unconsolidated sedimentary aquifers that have higher clay content are more susceptible to land subsidence, while aquifers formed of consolidated rocks, such as carbonate rocks, sandstone or basalt, have very little compaction.

Subsidence can be inelastic (permanent), and elastic (temporary) based on the preconsolidation history of the sedimentary layer. The lowest hydraulic head experienced in a sedimentary layer is defined as the preconsolidation head and corresponds to the highest effective stress experienced at that location. When the hydraulic head drops below this level, inelastic deformation occurs and subsidence becomes permanent[44–47]. Inelastic subsidence is of particular concern as layers undergoing such deformation do not rebound, even after the aquifer recharges. In contrast, sediments undergoing elastic subsidence return to their previous state with natural or managed groundwater recharge. At the depths and pressures of typical aquifer systems, sands do not experience inelastic deformation, and the inelastic deformation is essentially limited to clays[46]. The relationship between groundwater pumping and land subsidence can be expressed by the following equation[42] -

$$\Delta b = \Delta h S_{sk} b_0 \quad (1)$$

where Δh is the change is hydraulic head (h) due to pumping, b_0 is the thickness of the aquifer experiencing the change in head, S_{sk} is the skeletal specific storage and Δb is the change in thickness of the aquifer, which results in surface deformation. For weaker sediments like clay, S_{sk} has higher values than stronger sediments. Therefore, subsidence (Δb) will be greater in a clay dominated aquifer than a sand-gravel aquifer if equal thickness (b_0) of both aquifers are experiencing similar change in head (Δh). The skeletal specific storage has elastic and inelastic components referred to as elastic skeletal specific storage (S_{ske}) and inelastic skeletal specific storage (S_{skv}). The S_{ske} and S_{skv} values are typically on the order of 10^{-5} and $10^{-3}\ m^{-1}$, respectively[47,48]. Since the S_{skv} value is roughly two orders of magnitude higher than the S_{ske} value[49] and only clays deform inelastically, clay has the dominant signal during inelastic subsidence[48].

Inelastic subsidence in a confined aquifer is associated with a significant time lag; therefore, the total change in aquifer storage resulting from over-pumping is not reflected in the initial values of land subsidence. When an aquifer is pumped, high permeability sediments like sand respond quickly to groundwater pumping, and the resulting deformation is small compared to clay[47]. In the later stage, clay layers start to release water into the dewatered sand pores, though the water storage of the total aquifer system remains unchanged. Pore water pressure in clay drops, increasing the effective stress followed by the collapse and rearrangement of clay particles and as a result, significant deformation. This delayed drainage time can range from several weeks to tens of years[46,50,51].

Supplementary Note 2: Leave-One-Area-Out Accuracy Test Classification Criteria

The subsidence probability from the Leave-One-Area-Out Accuracy (LOAO) Test outcomes for each training area were evaluated. A probability of 40% or more of subsiding >1cm/year was chosen as the threshold value for categorizing the results using the polygon boundary extent of each training region (Supplementary Fig. 14 (a, c, e) show respective polygon extents). A 'satisfactory' accuracy was assigned if the number of pixels with >40% subsidence probability was higher than the number of subsidence pixels observed in the training dataset that was held out within the extent of the polygon. Results for areas where some subsidence probability of >40% was observed, but the number of pixels was fewer than what was observed in the training dataset, were considered 'acceptable', as the model was able to predict some high subsidence signatures over that area without the model being trained over that area. If these two criteria were not met, LOAO test performance was assigned 'not satisfactory' for that region. There were seven regions in the original training dataset where subsidence values all belonged to the < 1cm/year class. These regions were classified as 'satisfactory' if <1 cm/year subsidence was predicted on more than 85% area of that region by the respective model. For example, in San Luis Valley, Colorado training data consisted of only <1 cm/year class and the model in LOAO test without this training data predicted only ~5.2% pixels with higher subsidence probability. Therefore, the model's predictive power for this region was considered 'satisfactory'.

Supplementary Methods

Supplementary Method 1: Processing Input Hydrologic and Land Use Datasets

Input variables (predictors) of this model include remotely sensed and model-based global gridded datasets, within the geographic boundary of -180° W, -60° S, 180° E, 90° N, that are proxies of principal hydrologic, geologic, and anthropogenic processes that drive land subsidence. The variables were integrated in a supervised machine learning model known as random forests. Random forests is a decision-tree based algorithm that can establish nonlinear relationships between predictors and a response variable to generate a final model prediction[2,52].

Supplementary Table 1 shows a list of all input datasets used in the model along with their original spatial resolution and sources. Many of these datasets were downloaded using the Google Earth Engine[53] (GEE) platform while some datasets, such as irrigated land use, major rivers, sediment thickness, were downloaded from sources provided by their developers. The timeline 2013-2019 was considered as the temporal window for aggregating (aggregation method mentioned in Supplementary Table 1) time varying datasets prior to downloading from GEE. To integrate data in the machine learning model, all datasets needed to be in a uniform format under the same coordinate system, pixel size, and extent. The downloaded datasets were projected in the WGS 1984 geographic coordinate system. Depending on the original resolution, the datasets were downscaled/upscaled to a resolution of 0.02 deg (~2 km) using the 'nearest neighbor' algorithm to achieve a uniform grid size.

High-resolution (~4 km) precipitation, soil moisture, and evapotranspiration (ET), data were obtained from TerraClimate monthly climate and climatic water balance datasets[5]. Despite availability of finer spatial resolution remotely sensed ET data, such as MODIS global terrestrial

evapotranspiration data[54], ET data from Terraclimate was selected for its global coverage, including arid regions like Southern Africa, Western Asia, Central Australia where other datasets, like MODIS, have no coverage.

To provide irrigated area information to the model, we collected and tested several datasets. Siebert et al.[55] developed a coarse-resolution dataset of area equipped for irrigation (AEI) and area actually irrigated (AAI) for both surface and groundwater sources, using census information (e.g., country report, online database, Food and Agriculture Organization database, international organization report). Due to unavailability of AAI data in most countries/regions, some assumptions were made to extract AAI information from the AEI data in this dataset. Moreover, the dataset was not validated with in-situ information. Thenkabail et al.[56] developed the first remote sensing-based irrigated and rainfed crop dataset at 10-km resolution and classified between groundwater and surface water irrigation at some length. Both Siebert et al.[55] and Thenkabail et al.[56] datasets were tested in the model, but they didn't perform well, possibly because of their coarse resolution or lack of in-situ validation in areas with less data. To provide high-resolution information to the model, we chose irrigated area data (spatial resolution ~1 km) from Meier et al.[6]. This dataset was developed by downscaling data from Siebert et al.[55] using remote sensing information and was validated with ground-truth data. The dataset performed well in our model. Though the dataset cannot distinguish between groundwater and surface water irrigation, the model gets the sense of groundwater irrigation from this data with the help of other predictor variables.

A gridded population dataset of ~1 km resolution[7] was included in the model to capture subsidence in populated areas occurring from aquifer pumping. A gaussian filter (kernel filtering window size 3σ pixels) was applied to both of the irrigated area and population datasets to add a smoothing effect that accounts for groundwater depletion in regions adjacent to aquifer pumping and to

remove noise. The gaussian filter normalized the datasets within an interval range of 0 to 1, where larger values represent higher density of respective land use class and vice versa. Additional datasets included as predictors in the model were aridity index[13], MODIS-derived Normalized Difference Water Index (NDWI)[12], and Enhanced Vegetation Index (EVI)[11], and SRTM-derived percent slope. A vector dataset of major global rivers[10] was processed into a gridded format, representing distance from major global rivers, and incorporated in the model as well.

Supplementary Method 2: Developing "Presence of Confining Layer" Dataset

As the presence of a confining layer has a pronounced impact on the relationship between pumping and subsidence, a dataset indicating the likely presence or absence of a confining layer was produced as part of this study (Supplementary Fig. 11). This confining layer dataset was derived based on the depositional environment of basins, and was produced using a globally available digital elevation model (DEM) from the Shuttle Radar Topography Mission (SRTM)[57]. The premise used in creating this dataset is that regions that are likely to have had lacustrine or oceanic depositional environments over the past several hundred thousand years are also likely to have extensive clay layers, which confine the aquifer and result in more subsidence from groundwater withdrawals.

Regions likely to have extensive oceanic depositional environments were determined by identifying all DEM pixels with an elevation greater than 0 and less than 25 m. Since climate studies indicate that the sea level has fluctuated up to 25 m above the current sea level in the Holocene[58,59], this is likely the highest elevation that extensive fine-grained deposits due to an oceanic depositional environment would have occurred.

Regions likely to have extensive lacustrine depositional environments were determined by identifying existing basins with partial or full topographic depressions. Even if these regions do not currently have lakes, they are likely to have had lakes in their geologic past[60]. To identify these regions, we first selected all pixels with a slope of less than 5 m per km. These pixels were then polygonized. All polygons with an area of less than 20 km^2 were removed. A buffer of 20 km around each polygon was then created, and the 25th percentile of elevation was computed in the surrounding buffer. Basins whose mean elevation was lower than the 25th percentile of the surrounding buffered region were considered to be either closed basins or semi-closed basins,

which were likely to have had surface water supply as well as surrounding topography to block surface water from flowing out, and thus form a lake, sometime in their geologic past.

The two depositional environments (lacustrine confined and marine confined) produced were combined to form a single layer with a 1 where a confining layer was likely present, and a 0 where a confining layer was likely not present.

We validated our confining layer model using major aquifers in the United States as defined by the Groundwater Atlas of the United States[15], because this provides an extensive, geo-referenced map of aquifers with major confining layers. Supplementary Table 2 shows a summary of each major aquifer, along with the percent area of each aquifer that is estimated to have a confining layer present based on the methods outlined above. While there is some variance in the ability of our model to accurately estimate where confining layers are present, the average percent area with a confining layer present from our estimate is 22% over aquifers that have been mapped to have extensive confining layers, and 1% in areas that have been mapped to not have extensive confining layers. These results support our method, as we do not expect aquifers with extensive confining layers present to be 100% covered by a confining unit, nor do the reports from the USGS (2000)[15] suggest this to be the case. Rather, these aquifers have extensive confining layers that cover a significant portion of the basin, as indicated by our results. Note also that the three most extensively pumped aquifers in the United States (the Central Valley aquifer system, the Mississippi River Valley alluvial aquifer, and the High Plains aquifer[61]) are accurately characterized by our method. One final validation of our approach is that our subsidence model identifies the presence of a confining layer as an important predictor of total subsidence (Supplementary Figure 8), which is expected based on subsidence theory[46].

In spite of the general good agreement between mapped confining layer presence and our estimates, there are some discrepancies. We note that this, in addition to discrepancies present in other global datasets used in our model, contributes to overall model uncertainty.

Supplementary Method 3: Assembling Land Subsidence Dataset for Model Training

Small Baseline Subset (SBAS) InSAR time series analysis was conducted to estimate the average vertical subsidence rate. All Sentinel-1 data available for the research area between 2014 and 2020 were used to construct the cumulative deformation map. The initial SBAS networks of interferograms were built first, and they were constrained by baseline thresholds for both time and space. The baseline thresholds are often set arbitrarily with the goal of increasing interferometric pairs while minimizing spatial and temporal decorrelation. We used 200 m and 200 days, respectively, as the spatial and temporal baselines thresholds. In order to eliminate the topographic phase component and resolve the deformation, the Differential InSAR approach (DInSAR) was then applied to the chosen interferometric pairs. The co-registration process, interferogram generation, removal of phases related to the flat earth and topography, filtering, and unwrapping analysis were the first steps in the workflow carried out using GMTSAR software[62]. Having completed the unwrapping phase, the next step was to use SBAS analysis to obtain the time series deformation maps for the region. To reduce the impact of decorrelation effect, we reconstructed a modified SBAS network which only contained the interferometric pairs that were highly correlated within the targeted area. Using the described workflow, the SBAS network was created for each pixel separately. Only interferograms with coherence values greater than the SBAS analysis threshold (0.2) were considered. This raised the density of Persistent Scatterer (PS) points while also increasing the number of SBAS interferometric pairs. Additionally, having more interferograms allowed us to reduce temporally uncorrelated effects like tropospheric errors. The above workflow was implemented on each ascending and descending tracks to produce displacements along line of sight (LOS). LOS velocities were further decomposed into vertical

and horizontal components using measurements from ascending and descending imaging geometries.

Processed, georeferenced vertical subsidence data over California and Arizona in the USA were collected from the California Department of Water Resources and from the Arizona Department of Water Resources. Similarly, processed, georeferenced vertical subsidence data over 15 regions of Europe were collected from European Ground Motion Service (EGMS). Considering the substantial computational effort required in processing InSAR data, and the challenges associated with interpreting the principal subsidence cause to be related to groundwater, data was also collected from secondary sources. These sources consist of groundwater studies that used InSAR or GNSS information to determine aquifer vertical deformation. Regions where secondary sources were used include China, Indonesia, Iran, Turkey, USA and Vietnam. A comprehensive list of these training data sources is provided in the Supplementary Table 3.

For collecting secondary data, a database of almost 300 subsidence studies around the world was developed. The database was filtered for studies that analyzed InSAR data to study subsidence solely associated with groundwater pumping. All temporally dynamic input datasets were aggregated over a time window between 2013-2019. However, this timeline could not be coherently followed for processing/extracting each land subsidence dataset due to lack of data availability and quality in a particular region. In this study, the primary goal is to map average vertical land subsidence due to groundwater withdrawal. It was assumed that average vertical land subsidence remains approximately constant over an area if groundwater usage pattern remains the same. Therefore, the timeline of accumulating subsidence data was extended further back based on the availability of quality data. For example, average land subsidence data was extracted for the timeline 2003-2010 for Beijing, China from Chen et al.[18] and for the timeline 2014-2017 for

Mashhad, Iran from Khorrami et al.[29]. The processing timeline of subsidence data for each training region has been reported in Supplementary Table 3. To extract data from filtered research articles, images of average vertical deformation maps (digital, but not georeferenced, images) were georeferenced using ArcGIS tools. An ArcGIS built-in tree-based algorithm was used to extract deformation information from the georeferenced maps. If deformation was reported in the Line of Sight (LOS) direction, it was converted to vertical values using the satellite incidence angle, considering the horizontal component of subsidence negligible following the one-dimensional consolidation theory[44]. Such consideration generated some uncertainty to the training dataset, which has been discussed in the Supplementary Discussion 3. The extracted data were classified into three classes: <1 cm/year subsidence, 1-5 cm/year subsidence, and >5 cm/year subsidence. Subsidence data collected from research articles are referred to as georeferenced subsidence data in this study; however, these data are based on InSAR processing. Vertical land deformation data for coastal regions around the world was integrated from Shirzaei et al.[41], a coastal subsidence study based on data from GNSS networks, and classified into the subsidence categories. Out of nearly 3000 samples of this dataset, 98% fell into the <1 cm/year class. For this global study, <1 cm/year subsidence is considered as negligible to no subsidence class while the other classes represent medium to significant subsidence. It should be noted that subsidence of <1 cm/year values can be significantly damaging for coast-side regions due to the impact of climate change and resulting sea level rise, but predicting this level of subsidence was out of the scope of this study.

Supplementary Discussion

Supplementary Discussion 1: Input Variable Importance

To determine how the random forests model evaluated the input variables in predicting land subsidence, the importance of individual parameters (Supplementary Fig. 8) was assessed using permutation importance[2], a method that randomly permutes a variable to assess its importance in the model. Here, permutation importance is evaluated on the test dataset, considering that importance evaluation on the train dataset will be too optimistic as the model is already trained on that data. Permutation importance runs the model multiple iterations and shuffles an input variable's value in each iteration to evaluate relative change in model performance[2]. For variables that cause the most change in relative model accuracy (macro-F1 in this case) due to the permutation are considered the most important predictors. Permutation importance is a robust method in determining predictor importance, especially in cases of multicollinearity and cardinality[63,64], and performs reasonably better than node purity-based Gini importance estimates[65]. Based on permutation importance, the model considered soil moisture, confining layers, normalized clay indicator, normalized irrigated area density, and river distance as the most important variables among the 13 variables used. Interpretation of variable importance has been discussed in detail in Supplementary Discussion 2.

Supplementary Discussion 2: Interpreting Important Variables of the Model

This section discusses how the model interpreted the most important variables (predictors) based on the variable importance (Supplementary Figure 8) and partial dependence plots (Supplementary Figure 4 to 7).

Soil Moisture: Soil moisture acts like a ‘memory’ keeping track of the interaction between hydrologic fluxes e.g., precipitation and irrigation activity in soil. Low soil moisture from a land surface model that does not account for irrigation, such as the one used in this study, indicates high irrigation water demand in croplands. High groundwater irrigation can lead to subsidence if certain geologic conditions are met. Partial Dependence plot (PDP) of soil moisture and normalized irrigated area density (Fig. 6(a)) shows that the model has a higher probability of subsidence if there is low soil moisture and high irrigated area density.

Irrigation demands more groundwater in arid and semi-arid regions where precipitation is lower than evapotranspiration and surface water sources are scarce, which increases the possibility of subsidence. The model predicts higher subsidence probability in regions with low precipitation and soil moisture (Fig. 6(b)). Thus, soil moisture is a variable that is connected to other hydrologic fluxes and also works as an indicator of irrigation activity. For these reasons, it is regarded as the most important predictor of subsidence by the model (Fig. 8).

Soil moisture is not a direct driver of land subsidence, rather an indicator of hydrologic state that might lead to subsidence if other principal drivers of subsidence are in a favorable condition to let subsidence happen. Modeled soil moisture is in essence acting as a proxy for water availability in the soil column. To analyze whether low modeled soil moisture in the model by itself can result in a prediction of subsidence, or whether a combination of subsidence drivers (confining layer,

normalized clay indicator, normalized irrigated area density) are needed to predict subsidence, we performed a soil moisture sensitivity analysis test. We used the trained model with the 13 predictor variables to perform the test, where three scenarios were created based on the predictor variables values of soil moisture, confining layers, normalized clay indicator, and normalized irrigated area density (hereby denoting as ‘Variables of Interest’ (VOI)), while the other variables values came arbitrarily from the selection criteria of the VOI (detail in Supplementary Table 7). We used the validation dataset (unseen by the model during training phase) during this analysis to ensure that the trained model does not explicitly know the data but can reflect its learning from the training phase on this dataset.

The first scenario consists of low soil moisture, no confining layer, low normalized clay indicator, and no irrigated area, where the goal is to observe whether the model predicts subsidence only based on low soil moisture, which is a proxy of low water availability. Supplementary Fig. 9(a) shows that the model predicts no subsidence in this combination of VOI, meaning low soil moisture alone is not enough to predict subsidence even though soil moisture is listed as the most important predictor.

In the second scenario, we chose low soil moisture with the presence of confining layers, high normalized clay indicator, and high irrigated area density to observe whether the model predicts subsidence in combination of low soil moisture with direct drivers of subsidence. Supplementary Fig. 9(b) shows that our model predicts moderate to high subsidence the majority of the time under this scenario, meaning that for the model to predict subsidence, the geologic and water use variables need to be indicating subsidence along with low soil moisture.

Finally, in the third scenario, there is high soil moisture (which would mean generally the likelihood of subsidence is lower), confining layers present, high normalized clay indicator, and

high irrigated area density. Supplementary Fig. 9(c) shows that our model predicts no subsidence most of the time in this scenario but there are still a significant number of cases (roughly one third) where moderate to high subsidence is predicted. This indicates that even with high soil moisture the model predicts subsidence in some cases if the other three VOI are in favorable condition to subsidence.

The soil moisture sensitivity analysis test demonstrates that soil moisture, despite being the most important predictor in the variable importance plot, is not the sole indicator of subsidence, rather a combination of low to high soil moisture, presence of confining layers, high normalized clay indicator, and high irrigated area density is required for the model to predict subsidence.

Confining Layers: Our model considers 'Confining Layers' as one of the most important predictors (Fig. 8) of subsidence. The confining layer dataset represents the likely presence of extensive clay layers in regions that are likely to have lacustrine or oceanic depositional environments over the past several hundred thousand years. Aquifers confined by these extensive clay layers can undergo subsidence due to groundwater withdrawal; therefore, 'Confining Layers' is considered as one of the key drivers of subsidence of higher magnitude. Fig. 5 shows that subsidence probability increases (for 1-5 cm/year and >5 cm/year subsidence classes) with the presence of confining layer. The role of fine-grained confining layers in subsidence has been discussed in detail in Supplementary Note 1.

Normalized Clay Indicator: The **'**Normalized Clay Indicator' dataset supplements the information provided by the 'Confining Layers' dataset, except it is a continuous variable ('Confining Layers' is a binary variable) indicating the relative amount of clay in the subsurface. Fig. 4(a) and 4(b) show how the probability of subsidence increases with the increasing value of normalized clay indicator (probability slightly decreases in case of >5 cm/year class beyond 95

percentile value). The role of clay in subsidence has been discussed in detail in Supplementary Note 1.

Normalized Irrigated Area Density: Groundwater irrigation is a direct driver of land subsidence, as excessive pumping leads to subsidence. Therefore, irrigated area density is considered as one of the most important variables to predict land subsidence by the model. The model shows high subsidence prediction with increased irrigated area density (Fig 4). Though irrigated area density does not directly represent the intensity of groundwater irrigation, combinations of irrigated area density with other variables, such as distance from river distance (Fig. 7(a)), normalized clay indicator (Fig. 2 of main text) tells the model where groundwater irrigation activity is significant. Thus, irrigated area density, in combination with other variables, serves as a proxy of groundwater irrigation density in the model and is regarded as one of the key variables.

River Distance: The ‘River Distance’ dataset was included in the model to represent the distance from major surface water sources. The model considers this variable as one of the key variables to predict subsidence (Fig. 8). Plots in Fig. 7 show how river distance plays an important role in subsidence prediction despite not being a direct driver of subsidence. Groundwater is the main source of irrigation water and water supply in agricultural lands and populated areas distant from surface water sources. In such regions, excessive groundwater withdrawal can lead to subsidence. For this reason, the river distance dataset is regarded as a key predictor by the model.

Supplementary Discussion 3: Study Uncertainties

Machine learning model performance is related to the quality and quantity of the training data. Training data used in this model has a reasonable number of observations, although class imbalance restricts the model's ability to predict minority classes (1-5 cm/year and >5 cm/year) to some extent. A significant number of training data was extracted from InSAR-based research articles (denoted as secondary Sources) on subsidence. This process involved georeferencing digital subsidence maps from the articles and accurately segmenting the extracted deformation rates into three classes. Both processes are based on human perception and are prone to some level of uncertainty. A georeferencing residual value below 0.004 degrees (~400 m, where our pixel size was 2 km) was maintained in all cases to ensure uniformity between the extracted data except for the Tigris Euphrates basin, Iraq, where georeferencing residual exceeded the threshold value for some points, but this dataset was checked with basemap to ensure that the overall georeferencing quality was good. Note that we pre-screened the articles for groundwater withdrawal-associated subsidence only so that subsidence from other sources like significant tectonic deformations are not included in training data. For studies that reported average subsidence values in the Light of Sight (LOS) direction, the LOS values were converted to vertical values using the satellite incidence angle, assuming that the horizontal deformation is negligible compared to the vertical deformation for those aquifers[44]. Such an assumption can cause an overestimation of vertical subsidence near the boundary of subsiding regions[25] and well locations[44]. However, this technique has been widely used in many studies[25,46,66], especially in cases where vertical displacement could not be resolved due to the lack of deformation data from both ascending and descending satellite tracks.

InSAR processing is the primary source of land deformation data in the research articles from which training data was extracted. Additionally, InSAR data were directly processed over some regions to form the model's training dataset. Data processed from InSAR have uncertainties from atmospheric disturbance[17,67], irregularity in surface scattering[68], and tropospheric hindrance[69]. The InSAR data processed by the authors and many datasets from secondary sources have accounted for these uncertainties in their processing steps. In regions where we directly processed InSAR, our approach employs InSAR time series analysis using a large number of interferometric pairs in a small baseline network (SBAS), including all large temporal baselines, to reduce the effects of these sources of uncertainty. This approach allows us to detect the persistent and significant land elevation changes that are not likely to be affected by these sources of uncertainty. For example, the effects of plowing of fields, crop growth, and soil moisture variations can be temporally uncorrelated, meaning that they may vary over time and are not consistently present in the same way over a given period. By considering both large and small temporal baselines, we are able to more accurately detect persistent and significant land elevation changes that are not likely to be affected by sources of uncertainty. Uncertainties were quantified over regions where we processed InSAR or collected the data from other sources. We estimated an uncertainty range between ~1.4 to 7.2 mm, using a spatial statistical method called the variogram[70], for Colorado, USA; Hebei & Hefei, China; Qazvin, Iran, regions where we directly processed InSAR. We also processed InSAR data over Quetta Valley, Pakistan, which has a relatively higher error of ~2.2 cm. The Central Valley, California data has a reported uncertainty[71] ranging from 0.5 to 2 cm. The InSAR data over Arizona, United States has an uncertainty of ~1 cm[72]. The European Ground Motion Service reports errors between 1 to 2 mm for InSAR datasets over the 15 regions in Europe. Overall,

uncertainties in our InSAR training dataset generally fall between the typical ranges of error, which is reported to be on the order of 5 mm to 1 cm by literature[25,73].

The subsidence values in the InSAR-derived training data represent the average deformation velocity, which was computed as the best-fit linear subsidence rate over the processing periods. Subsidence is a non-linear process that can vary depending on the geologic setting, hydraulic head, etc. The linear rate cannot characterize the seasonal change in subsidence and fluctuation of its values due to any reason. Despite that, the prime objective of this study is to quantify/detect long-term subsidence, and the average linear subsidence rate is a reasonable approximation for this [74,75].

Point-scale Global Navigation Satellite System (GNSS)-based coastal vertical land subsidence data[41] were pixelated to 2-km resolution, assuming that vertical deformation in an entire pixel is the same as the point-scale, and added to the model to supplement the training land subsidence data. This assumption can add uncertainty in pixels where the subsidence rate is not limited to a single class that the pixel-based estimate denotes. Moreover, Shirzaei et al.[41] reports that this dataset accounts for a lower bound of subsidence values in some regions. Though the model predicts subsidence in ranges of magnitude, these lower bound estimates can incorporate some uncertainty in the model predictions and underpredict subsidence, especially in regions where actual subsidence is near the model's class boundary values (e.g., near 1 or 5cm/year).

Gridded datasets used in this study as input variables have inherent uncertainties from their sources. Remotely sensing based estimates are bound by their sensor's limitation. Sometimes post-processing, filtering, and statistical methods are applied on remote sensing datasets to refine the data or to develop a new dataset from multiple remote sensing estimates, such as land use classification. Such techniques transfer the parent dataset's uncertainties to the new dataset formed. For example, TerraClimate climatic estimates inherit uncertainties from their parent

datasets. Water balance components of TerraClimate comes from a 1-D land surface model and limitations of the model assumptions can add uncertainties to the resulting estimates[5]. The 'confining layers' dataset was developed during this study and used as a model predictor. Discussion on its creation, validation, and uncertainty has been highlighted in the Supplementary Method 2. Moreover, we feature-engineered the 'normalized clay indicator' variable using datasets of percent clay content data at 200 cm depth and average unconsolidated material thickness (detail in main manuscript methods section). The percent clay content dataset is an estimate of clay percentage only at 200 cm depth and is not a representative of the whole subsurface, thus, adding some uncertainty to the model. Despite that, merging it with average unconsolidated material thickness creates a proxy dataset that represents the presence of clay in the subsurface to some extent providing valuable information to the model.

Due to the unavailability of direct groundwater pumping estimate (which is one of the principal drivers of subsidence) at a global scale, we used irrigated area density (also population density) as a proxy variable to represent groundwater use intensity in our model. Some uncertainty is introduced into the model due to uncertainty in the source of this irrigation data (surface water or groundwater) as regions mapped as irrigated could be sourced from surface water. Our model overestimates subsidence in some irrigated regions, such as Albacete and Ciudad Real province, Spain, possibly due to this reason. Moreover, as irrigated area density does not directly represent values of groundwater pumping, the model overpredicts subsidence in some known groundwater-irrigated regions, such as the High Plains Aquifer. A transition from surface water to groundwater irrigation and increased groundwater pumping could result in future subsidence in such areas. Thus, mapped subsidence could indicate a susceptibility to future subsidence in some regions where the model overpredicts subsidence.

Supplementary Discussion 4: Subsidence Probability

To assess the confidence of the model in predicting subsidence, a probability map was generated (Supplementary Fig. 2 and 3) showing the probability of subsidence greater than 1 cm/year magnitude on a global scale. This map was generated by using the full ensemble of decision tree predictions to compute the fraction of decision tree predictions that estimated either 1-5 cm/year or >5 cm/year subsidence. The map demonstrates the confidence of our model that subsidence will occur. Some regions that did not show any predicted subsidence (Supplementary Fig. 2 and 3) (i.e., the majority vote, or most likely prediction from our model was <1 cm/year subsidence), still have some probability of subsidence (i.e., relatively few decision trees predicted subsidence). Regions with low model confidence may occur in regions that have little or no training data with comparable climate, geologic, or land use[72].

This probability map could also be interpreted as a subsidence susceptibility map. Some areas with moderate probability of subsidence that are not currently subsiding could subside if the conditioning factors like groundwater use increase. Overall, high subsidence probabilities around the world were observed in mostly agricultural and urban lands where groundwater dependency is significant. Probability values are high in regions where subsidence is currently happening or has been predicted to happen by the majority vote of our model.

In Asia, a vast region in North China Plain is significantly subsiding with more surrounding areas having some probability of subsidence. Similar susceptibility trends are observed in agricultural lands in Iran, Turkey, Syria in middle-Eastern Asia and some countries in central Asia. A 40-60% probability observed near the coasts of Bangladesh, Myanmar, and Taiwan (>60% probability regions are already subsiding in Taiwan) signify that these regions are vulnerable to subsidence, particularly if groundwater usage practices are not altered. Agricultural lands in Israel and coastal

regions in Vietnam, Thailand, Philippines, and Indonesia are also under high subsidence susceptibility (Supplementary Fig. 2 and 3). In North America, high subsidence probabilities in California, Arizona, and Texas in the USA, and in Mexico City, Mexico indicates already occurring deformation. A significant percentage of lands in the High Plains Aquifer region show high probability (40-60%) of subsidence. Built-up areas in central Mexico also appear to be under high subsidence likelihood.

In South America, parts of Argentina, near the coast, show a high probability of subsidence (Supplementary Fig. 2 and 3). Irrigated agriculture dataset from Meier et al.[6] shows irrigated croplands over this region. A large portion of the Murray-Darling basin in South-western Australia shows some probability of subsiding more than 1 cm/year, which aligns with the results found in a previous study[16]. In Europe, potential subsidence susceptibility exists in Spain, Italy, and Ukraine. In Africa, parts of Egypt, Morocco, Libya, Algeria, and Tunisia shows subsidence susceptibility (Supplementary Fig. 2 and 3).

Supplementary Discussion 5: Comparison with Global Studies

There have been very few studies on global groundwater storage change, and none to the authors' knowledge that evaluate deep confined or semi-confined aquifers. Our model predicts groundwater storage loss in deep aquifers in terms of land subsidence. Here, we compare the model's results with a global groundwater depletion study by Wada et al.[4]. This global groundwater depletion dataset was derived by estimating recharge at each pixel with a water balance model and estimating the groundwater withdrawals based on land use and available withdrawal datasets. The dataset does not distinguish between unconfined and confined groundwater storage loss, while our dataset is primarily an indication of confined groundwater storage loss. Supplementary Fig. 15 shows the comparison between two studies in major groundwater stressed regions.

In North China Plain (Supplementary Fig. 15(b)), similar groundwater storage loss patterns (>1 cm/year) are observed in both studies. In North America (Supplementary Fig. 15(a)), both studies predict aquifer loss in the Central Valley (California) and central Mexico. Wada et al.[4] estimates large depletion in the High Plains Aquifer (HPA) region in the US; however, our model only shows subsidence in small parts of this aquifer. In central Asia, our results show aquifer storage loss in Uzbekistan, Afghanistan, Turkmenistan, Kazakhstan, and Azerbaijan in contrast to Wada et al.[4]. Both models predict groundwater depletion over Iran, while Wada et al.[4]'s estimate covers a larger spatial extent than our study. Large groundwater depletion in India is estimated in Wada et al.[4], where our model predicts no significant subsidence.

The fundamental reason for most differences observed between these two datasets is that many regions of the world are experiencing substantial groundwater storage loss without experiencing significant subsidence. Most notably, large portions of northern India and the HPA, both of which have withdrawals primarily occurring in unconfined aquifers[76,77], show significant depletion in

Wada et al.[4] but little to no deformation in our study. In these primarily unconfined regions, the main mechanism for storage loss is drainage or desaturation of pores, which buffers the drop in head and mitigates subsidence[78]. Consolidation and the resulting subsidence is the primary mechanism for storage loss in confined aquifers. In regions with more confining aquifers, such as California, East Asia, and Iran, groundwater depletion causes subsidence of similar magnitude. It is noteworthy that even in these regions, there are portions of the aquifer, typically along alluvial fans near the borders of the basin, where the aquifer is unconfined, and thus depletion may occur without significant subsidence.

Some regions where our model predicted subsidence but no depletion was estimated by Wada et al.[4] could be related to differences in model resolution, or inaccuracies in withdrawal data used by Wada et al.[4]. The reported spatial resolution of the depletion data is 0.5 deg (~55 km), which is much coarser than our model resolution. This might also have an impact on the larger spatial extent reported by the study compared to our results in some places.

In addition, we compared our model results over 27 regions of Europe with European Ground Motion Service (EGMS)-provided vertical deformation data. The model's training subsidence data includes these 27 regions, though the data has been resampled (to 2 km) and reclassified (to model classes) before inclusion. Analysis shows that most of the areas have <1 cm/year subsidence if compared at 2-km scale. In Murcia, Spain and Po Delta, Italy, there are 1-5 cm/year predictions over some irrigated agricultural lands detected by both the model and EGMS data. Comparison between the model's 2-km prediction and EGMS's ~100m resolution data showed that there are some very localized >1 cm/year subsidence detected in the EGMS data (in <0.5% area over the 15 regions). Our model couldn't detect these localized (at less than 2-km scale) subsidence due to the model's native resolution of 2 km. Moreover, our model overpredicts subsidence over irrigated

lands in Albacete and Ciudad Real province, Spain where the EGMS data show <1 cm/year subsidence.

Comparison of our study result in North China Plain with a regional study[79] also found similar groundwater storage loss trends in that area by both studies. Future studies should be focused on regions where our model's prediction does not converge with other studies/data.

Supplementary References

1. Hastie, T., Tibshirani, R. & Friedman, J. *The Elements of Statistical Learning*. (Springer New York Inc., 2001).

2. Breiman, L. Random Forests. *Mach. Learn.* **45**, (2001).

3. IU Digital Science Center. Harp Random Forests. https://dsc-spidal.github.io/harp/docs/examples/rf/ (2013).

4. Wada, Y. *et al.* Global depletion of groundwater resources. *Geophys. Res. Lett.* **37**, 1–5 (2010).

5. Abatzoglou, J. T., Dobrowski, S. Z., Parks, S. A. & Hegewisch, K. C. TerraClimate, a high-resolution global dataset of monthly climate and climatic water balance from 1958-2015. *Sci. Data* **5**, 1–12 (2018).

6. Meier, J., Zabel, F. & Mauser, W. A global approach to estimate irrigated areas - A comparison between different data and statistics. *Hydrol. Earth Syst. Sci.* **22**, 1119–1133 (2018).

7. CIESIN. GPWv411: UN-Adjusted Population Density (Gridded Population of the World Version 4.11). (2018) doi:10.7927/H4F47M65.

8. Hengl, T. Clay content in % (kg / kg) at 6 standard depths (0, 10, 30, 60, 100 and 200 cm) at 250 m resolution. (2018) doi:10.5281/zenodo.1476854.

9. Pelletier, J. D. *et al.* Global 1-km Gridded Thickness of Soil, Regolith, and Sedimentary Deposit Layers. (2016) doi:10.3334/ORNLDAAC/1304.

10. GRDC. Major River Basins of the World / Global Runoff Data Centre, GRDC. 2nd, rev. ext. ed. Koblenz, Germany: Federal Institute of Hydrology (BfG). https://www.bafg.de/GRDC/EN/02_srvcs/22_gslrs/221_MRB/riverbasins_node.html (2020).

11. Didan, K. MODIS/Terra Vegetation Indices 16-Day L3 Global 250m SIN Grid V061 EVI. *NASA EOSDIS Land Processes DAAC* (2021) doi:10.5067/MODIS/MOD13Q1.061.

12. Vermote, E. MODIS/Terra Surface Reflectance 8-Day L3 Global 500m SIN Grid V061. *NASA EOSDIS Land Processes DAAC* (2021) doi:10.5067/MODIS/MOD09A1.061.

13. Trabucco, A. & Zomer, R. Global Aridity Index and Potential Evapotranspiration (ET0) Climate Database v2. (2019) doi:10.6084/m9.figshare.7504448.v3.

14. Farr, T. G. *et al.* The Shuttle Radar Topography Mission. *Rev. Geophys.* **45**, RG2004 (2007).

15. *Ground Water Atlas of the United States*. *Hydrologic Atlas 730* http://pubs.er.usgs.gov/publication/ha730 (2000) doi:10.3133/ha730.

16. Castellazzi, P. & Schmid, W. Interpreting C-band InSAR ground deformation data for large-scale groundwater management in Australia. *J. Hydrol. Reg. Stud.* **34**, 100774 (2021).

17. Higgins, S. *et al.* InSAR measurements of compaction and subsidence in the Ganges-Brahmaputra Delta, Bangladesh. *J. Geophys. Res. F Earth Surf.* **119**, 1768–1781 (2014).

18. Chen, M. *et al.* Imaging Land Subsidence Induced by Groundwater Extraction in Beijing (China) Using Satellite Radar Interferometry. *Remote Sens.* **8**, 468 (2016).

19. Higgins, S., Overeem, I., Tanaka, A. & Syvitski, J. P. M. Land subsidence at aquaculture facilities in the Yellow River delta, China. *Geophys. Res. Lett.* **40**, 3898–3902 (2013).

20. Dong, S., Samsonov, S., Yin, H., Ye, S. & Cao, Y. Time-series analysis of subsidence associated with rapid urbanization in Shanghai, China measured with SBAS InSAR method. *Environ. Earth Sci.* **72**, 677–691 (2014).

21. Zhou, L. *et al.* Wuhan surface subsidence analysis in 2015-2016 based on sentinel-1A data by SBAS-InSAR. *Remote Sens.* **9**, (2017).

22. Qu, F. *et al.* Land subsidence and ground fissures in Xi'an, China 2005-2012 revealed by multi-band InSAR time-series analysis. *Remote Sens. Environ.* **155**, 366–376 (2014).

23. Luo, Q., Perissin, D., Zhang, Y. & Jia, Y. L- and X-band multi-temporal InSAR analysis of tianjin subsidence. *Remote Sens.* **6**, 7933–7951 (2014).

24. Gebremichael, E. *et al.* Assessing Land Deformation and Sea Encroachment in the Nile Delta: A Radar Interferometric and Inundation Modeling Approach. *J. Geophys. Res. Solid Earth* **123**, 3208–3224 (2018).

25. Chaussard, E., Amelung, F., Abidin, H. & Hong, S. H. Sinking cities in Indonesia: ALOS PALSAR detects rapid subsidence due to groundwater and gas extraction. *Remote Sens. Environ.* **128**, 150–161 (2013).

26. Ge, L., Ng, A. H. M., Li, X., Abidin, H. Z. & Gumilar, I. Land subsidence characteristics of Bandung Basin as revealed by ENVISAT ASAR and ALOS PALSAR interferometry.

Remote Sens. Environ. **154**, 46–60 (2014).

27. Andaryani, S., Nourani, V., Trolle, D., Dehgani, M. & Asl, A. M. Assessment of land use and climate change effects on land subsidence using a hydrological model and radar technique. *J. Hydrol.* **578**, 124070 (2019).

28. Haghighi, M. H. & Motagh, M. Ground surface response to continuous compaction of aquifer system in Tehran, Iran: Results from a long-term multi-sensor InSAR analysis. *Remote Sens. Environ.* **221**, 534–550 (2019).

29. Khorrami, M., Abrishami, S., Maghsoudi, Y., Alizadeh, B. & Perissin, D. Extreme subsidence in a populated city (Mashhad) detected by PSInSAR considering groundwater withdrawal and geotechnical properties. *Sci. Rep.* **10**, 1–16 (2020).

30. Rateb, A. & Kuo, C. Y. Quantifying vertical deformation in the Tigris-Euphrates basin due to the groundwater abstraction: Insights from GRACE and Sentinel-1 satellites. *Water (Switzerland)* **11**, (2019).

31. Garg, S., Motagh, M., Indu, J. & Karanam, V. Tracking hidden crisis in India's capital from space: implications of unsustainable groundwater use. *Sci. Rep.* **12**, 1–17 (2022).

32. Chaussard, E., Wdowinski, S., Cabral-Cano, E. & Amelung, F. Land subsidence in central Mexico detected by ALOS InSAR time-series. *Remote Sens. Environ.* **140**, 94–106 (2014).

33. Cian, F., Blasco, J. M. D. & Carrera, L. Sentinel-1 for monitoring land subsidence of coastal cities in Africa using PSInSAR: A methodology based on the integration of SNAP and staMPS. *Geosci.* **9**, (2019).

34. Zoysa, R. S. De *et al.* The 'wickedness' of governing land subsidence: Policy perspectives

from urban southeast Asia. *PLoS One* **16**, 1–25 (2021).

35. Orhan, O., Oliver-Cabrera, T., Wdowinski, S., Yalvac, S. & Yakar, M. Land subsidence and its relations with sinkhole activity in karapınar region, turkey: A multi-sensor insar time series study. *Sensors (Switzerland)* **21**, 1–17 (2021).

36. Aslan, G., Cakir, Z., Lasserre, C. & Renard, F. Investigating subsidence in the Bursa Plain, Turkey, using ascending and descending sentinel-1 satellite data. *Remote Sens.* **11**, (2019).

37. Hung, W. C. *et al.* Monitoring severe aquifer-system compaction and land subsidence in Taiwan using multiple sensors: Yunlin, the southern Choushui river Alluvial fan. *Environ. Earth Sci.* **59**, 1535–1548 (2010).

38. Miller, M. M. & Shirzaei, M. Land subsidence in Houston correlated with flooding from Hurricane Harvey. *Remote Sens. Environ.* **225**, 368–378 (2019).

39. Minh, D. H. T., Van Trung, L. & Le Toan, T. Mapping ground subsidence phenomena in Ho Chi Minh City through the radar interferometry technique using ALOS PALSAR data. *Remote Sens.* **7**, 8543–8562 (2015).

40. Nguyen, M. *et al.* Assessment of long-term ground subsidence and groundwater depletion in Hanoi, Vietnam. *Eng. Geol.* **299**, 106555 (2022).

41. Shirzaei, M. *et al.* Measuring, modelling and projecting coastal land subsidence. *Nat. Rev. Earth Environ.* **2**, 40–58 (2021).

42. Fetter, C. W. *Applied Hydrogeology Fourth Edition*. *Applied Hydrogeology* (Pearson Education Limited, 2001).

43. Terzaghi, K. Erdbaumechanik (Introduction to Soil Mechanics). *Vienna: Franz Deuticke*

1943–1944 (1925).

44. Galloway, D. L. & Burbey, T. J. Review: Regional land subsidence accompanying groundwater extraction. *Hydrogeol. J.* **19**, 1459–1486 (2011).

45. Faunt, C. C., Sneed, M., Traum, J. & Brandt, J. T. Water availability and land subsidence in the Central Valley, California, USA. *Hydrogeol. J.* **24**, 675–684 (2016).

46. Smith, R. *et al.* Estimating the permanent loss of groundwater storage in the southern San Joaquin Valley, California. *Water Resour. Res.* **53**, 2133–2148 (2017).

47. Sneed, B. M. *Hydraulic and Mechanical Properties Affecting Ground-Water Flow and Aquifer- System Compaction , San Joaquin Valley , California*. *U. S. Geological Survey* (2001).

48. Smith, R. & Knight, R. Modeling Land Subsidence Using InSAR and Airborne Electromagnetic Data. *Water Resour. Res.* **55**, 2801–2819 (2019).

49. Faunt, C. C. *Groundwater availability of the Central Valley Aquifer, California*. (2009).

50. Chen, J., Knight, R., Zebker, H. A. & Schreüder, W. A. Confined aquifer head measurements and storage properties in the San Luis Valley, Colorado, from spaceborne InSAR observations. *Water Resour. Res.* **52**, 3623–3636 (2016).

51. Lees, M., Knight, R. & Smith, R. Development and Application of a 1D Compaction Model to Understand 65 Years of Subsidence in the San Joaquin Valley. *Water Resour. Res.* **58**, (2022).

52. Biau, G. Analysis of a Random Forests Model. *J. Mach. Learn. Res.* **13**, 1063–1095 (2012).

53. Gorelick, N. *et al.* Google Earth Engine: Planetary-scale geospatial analysis for everyone.

Remote Sens. Environ. **202**, 18–27 (2017).

54. Mu, Q., Zhao, M. & Running, S. W. MODIS Global Terrestrial Evapotranspiration (ET) Product (NASA MODIS Global Terrestrial Evapotranspiration (ET) Product (NASA MOD16A2/A3) Collection 5. NASA Headquarters MOD16A2/A3) Collection 5. NASA Headquarters. (2013).

55. Siebert, S., Henrich, V., Frenken, K. & Burke, J. Update of the digital global map of irrigation areas to version 5. *Rheinische Friedrich-Wilhelms-Universituy, Bonn, Ger. Food Agric. Organ. United Nations, Rome, Italy* 171 (2013) doi:10.13140/2.1.2660.6728.

56. Thenkabail, P. S. *et al.* Global irrigated area map (GIAM), derived from remote sensing, for the end of the last millennium. *Int. J. Remote Sens.* **30**, 3679–3733 (2009).

57. NASA Shuttle Radar Topography Mission (SRTM). *Shuttle Radar Topography Mission (SRTM) Global. Distributed by OpenTopography.* (2013) doi:10.5069/G9445JDF.

58. Lambeck, K., Rouby, H., Purcell, A., Sun, Y. & Sambridge, M. Sea level and global ice volumes from the Last Glacial Maximum to the Holocene. *Proc. Natl. Acad. Sci.* **111**, 15296–15303 (2014).

59. Miller, K. G. *et al.* The Phanerozoic Record of Global Sea-Level Change. *Science (80-.).* **310**, 1293–1298 (2005).

60. Blair, T. C. Tectonic and hydrologic controls on cyclic alluvial fan, fluvial, and lacustrine rift-basin sedimentation, Jurassic-Lowermost Cretaceous Todos Santos Formation, Chiapas, Mexico. *J. Sediment. Res.* **57**, 845–862 (1987).

61. Dieter, C. A. *et al. Estimated use of water in the United States in 2015*. *Circular*

http://pubs.er.usgs.gov/publication/cir1441 (2018) doi:10.3133/cir1441.

62. Sandwell, D., Mellors, R., Tong, X., Wei, M. & Wessel, P. Open radar interferometry software for mapping surface Deformation. *Eos, Trans. Am. Geophys. Union* **92**, 234–234 (2011).

63. Toloşi, L. & Lengauer, T. Classification with correlated features: Unreliability of feature ranking and solutions. *Bioinformatics* **27**, 1986–1994 (2011).

64. Altmann, A., Toloşi, L., Sander, O. & Lengauer, T. Permutation importance: A corrected feature importance measure. *Bioinformatics* **26**, 1340–1347 (2010).

65. James, G., Witten, D., Hastie, T. & Tibshirani, R. *An Introduction to Statistical Learning wth application in R*. (2013).

66. Castellazzi, P., Garfias, J., Martel, R., Brouard, C. & Rivera, A. InSAR to support sustainable urbanization over compacting aquifers: The case of Toluca Valley, Mexico. *Int. J. Appl. Earth Obs. Geoinf.* **63**, 33–44 (2017).

67. Reeves, J. A., Knight, R. & Zebker, H. A. An analysis of the uncertainty in InSAR deformation measurements for groundwater applications in agricultural areas. *IEEE J. Sel. Top. Appl. Earth Obs. Remote Sens.* **7**, 2992–3001 (2014).

68. Erban, L. E., Gorelick, S. M., Zebker, H. A. & Fendorf, S. Release of arsenic to deep groundwater in the Mekong Delta, Vietnam, linked to pumping-induced land subsidence. *Proc. Natl. Acad. Sci.* (2013).

69. Fattahi, H. & Amelung, F. InSAR bias and uncertainty due to the systematic and stochastic tropospheric delay. *J. Geophys. Res. Solid Earth* **120**, 8758–8773 (2015).

70. Lohman, R. B. & Simons, M. Some thoughts on the use of InSAR data to constrain models of surface deformation: Noise structure and data downsampling. *Geochemistry, Geophys. Geosystems* **6**, n/a-n/a (2005).

71. Farr, T. G. InSAR measurements of subsidence in the Central Valley, California from 2007 - present. in *Proceedings of EUSAR 2016: 11th European Conference on Synthetic Aperture Radar* 1–3 (2016).

72. Smith, R. & Majumdar, S. Groundwater Storage Loss Associated With Land Subsidence in Western United States Mapped Using Machine Learning. *Water Resour. Res.* **56**, (2020).

73. Zebker, H. A., Rosen, P. A. & Hensley, S. Atmospheric effects in interferometric synthetic aperture radar surface deformation and topographic maps. *J. Geophys. Res. Solid Earth* **102**, 7547–7563 (1997).

74. Bell, J. W., Amelung, F., Ferretti, A., Bianchi, M. & Novali, F. Permanent scatterer InSAR reveals seasonal and long-term aquifer-system response to groundwater pumping and artificial recharge. *Water Resour. Res.* **44**, (2008).

75. Chaussard, E., Bürgmann, R., Shirzaei, M., Fielding, E. J. & Baker, B. Predictability of hydraulic head changes and characterization of aquifer-system and fault properties from InSAR-derived ground deformation. *J. Geophys. Res. Solid Earth* **119**, 6572–6590 (2014).

76. Weeks, J. B. High Plains regional aquifer-system study. *US Geol. Surv. Circ.* **1002**, 31 (1986).

77. Jha, B. M. & Sinha, S. K. Towards better management of ground water resources in India. *QJ* **24(4)**, 1–20 (2009).

78. Bouwer, H. Land Subsidence and Cracking Due to Ground-Water Depletiona. *Ground Water* **15**, 358–364 (1977).

79. Gong, H. *et al.* Long-term groundwater storage changes and land subsidence development in the North China Plain (1971–2015). *Hydrogeol. J.* **26**, 1417–1427 (2018).
