# Supplementary material for: Global land subsidence mapping reveals widespread loss of aquifer storage capacity
Source: Nat Commun. 2023 Oct 4;14:6180. doi: 10.1038/s41467-023-41933-z (PMC10550978; doi:10.1038/s41467-023-41933-z)
Supplement: Supplementary file 2 — Peer Review File [file 41467_2023_41933_MOESM2_ESM.pdf]

nature portfolio

Peer Review File

Global Land Subsidence Mapping Reveals Widespread Loss of Aquifer Storage Capacity

Open Access This file is licensed under a Creative Commons Attribution 4.0 International License, which permits use, sharing, adaptation, distribution and reproduction in any medium or format, as long as you give appropriate credit to the original author(s) and the source, provide a link to the Creative Commons license, and indicate if changes were made. In the cases where the authors are anonymous, such as is the case for the reports of anonymous peer reviewers, author attribution should be to 'Anonymous Referee' followed by a clear attribution to the source work. The images or other third party material in this file are included in the article's Creative Commons license, unless indicated otherwise in a credit line to the material. If material is not included in the article's Creative Commons license and your intended use is not permitted by statutory regulation or exceeds the permitted use, you will need to obtain permission directly from the copyright holder. To view a copy of this license, visit http://creativecommons.org/licenses/by/4.0/.

Editorial Note: This manuscript has been previously reviewed at another journal that is not operating a transparent peer review scheme. This document only contains reviewer comments and rebuttal letters for versions considered at Nature Communications.

Reviewers' Comments:

Reviewer #1:
Remarks to the Author:
.

Reviewer #2:
Remarks to the Author:
I am satisfied with the additional tests and explanations that the authors have included in the latest version of the manuscript in order to address my comments. I recommend that the manuscript be accepted in its current form.

Matt Rodell
NASA/GSFC
